# Identifying Loci Contributing to Natural Variation in Xenobiotic Resistance in *Drosophila*

**DOI:** 10.1371/journal.pgen.1005663

**Published:** 2015-11-30

**Authors:** Michael A. Najarro, Jennifer L. Hackett, Brittny R. Smith, Chad A. Highfill, Elizabeth G. King, Anthony D. Long, Stuart J. Macdonald

**Affiliations:** 1 Department of Molecular Biosciences, University of Kansas, Lawrence, Kansas, United States of America; 2 Division of Biological Sciences, University of Missouri, Columbia, Missouri, United States of America; 3 Department of Ecology and Evolutionary Biology, University of California Irvine, Irvine, California, United States of America; 4 Center for Computational Biology, University of Kansas, Lawrence, Kansas, United States of America; North Carolina State University, UNITED STATES

## Abstract

Natural populations exhibit a great deal of interindividual genetic variation in the response to toxins, exemplified by the variable clinical efficacy of pharmaceutical drugs in humans, and the evolution of pesticide resistant insects. Such variation can result from several phenomena, including variable metabolic detoxification of the xenobiotic, and differential sensitivity of the molecular target of the toxin. Our goal is to genetically dissect variation in the response to xenobiotics, and characterize naturally-segregating polymorphisms that modulate toxicity. Here, we use the *Drosophila* Synthetic Population Resource (DSPR), a multiparent advanced intercross panel of recombinant inbred lines, to identify QTL (Quantitative Trait Loci) underlying xenobiotic resistance, and employ caffeine as a model toxic compound. Phenotyping over 1,700 genotypes led to the identification of ten QTL, each explaining 4.5–14.4% of the broad-sense heritability for caffeine resistance. Four QTL harbor members of the cytochrome P450 family of detoxification enzymes, which represent strong *a priori* candidate genes. The case is especially strong for *Cyp12d1*, with multiple lines of evidence indicating the gene causally impacts caffeine resistance. *Cyp12d1* is implicated by QTL mapped in both panels of DSPR RILs, is significantly upregulated in the presence of caffeine, and RNAi knockdown robustly decreases caffeine tolerance. Furthermore, copy number variation at *Cyp12d1* is strongly associated with phenotype in the DSPR, with a trend in the same direction observed in the DGRP (*Drosophila* Genetic Reference Panel). No additional plausible causative polymorphisms were observed in a full genomewide association study in the DGRP, or in analyses restricted to QTL regions mapped in the DSPR. Just as in human populations, replicating modest-effect, naturally-segregating causative variants in an association study framework in flies will likely require very large sample sizes.

## Author Summary

A striking feature of biology is that within populations there is substantial interindividual phenotypic variation for traits of biomedical significance. Some fraction of this variation is due to environmental factors, but for many traits segregating genetic differences contribute significantly to phenotypic variation. Elucidating those causative sequence changes that result in complex trait variation is of central importance to biology, and requires the coordinated use of multiple approaches. Here we employ a multi-level strategy to dissect genetic variation in caffeine resistance in *Drosophila melanogaster*, leveraging powerful genetic screening in a multiparental mapping panel, a genomewide association study, high-throughput RNA sequencing, and gene knockdowns using RNA interference. We identify several short genomic regions that collectively explain a substantial portion of the heritable variation for caffeine resistance, and find that several of these regions harbor members of known detoxification enzyme families. One such gene—*Cyp12d1*—shows increased expression on exposure to caffeine, and experimentally reducing gene expression leads to a reduction in caffeine resistance. We additionally show that variation in the number of copies of *Cyp12d1* is positively associated with resistance. These compelling lines of evidence imply that structural variation at this gene causally contributes to xenobiotic resistance in *Drosophila*.

## Introduction

Living organisms are subjected to a barrage of toxic compounds, or xenobiotics, in their environment and their diet. Animals are frequently exposed to toxins produced by potential prey and/or plant hosts as chemical defenses [1, 2], and are increasingly subject to pressures from human activity, such as pollution and the application of pesticides. Humans themselves are additionally exposed to an array of xenobiotics throughout their lives in the form of pharmaceuticals [3]. Given the evolutionary, agricultural, and medical relevance of the response and resistance to toxins, dissecting the genetic factors responsible for xenobiotic metabolism is essential.

Some of the best understood cases of xenobiotic response mechanisms come from insect populations or species that are able to withstand pesticides or toxic host chemical defense compounds. In some cases, certain individuals are simply insensitive to the xenobiotic due to a change in the structure, and therefore function, of the target of the toxin. For example, the Monarch butterfly (*Danaus plexippus*) is resistant to cardenolides, a class of secondary metabolites toxic to most animals, produced by their milkweed host plant. This resistance is due to at least one amino acid change in the Monarch Na^+^, K^+^-ATPase gene that prevents dietary cardenolides binding to the protein, making the protein insensitive to cardenolide inhibition [4, 5]. In many other cases, prior to reaching its target, the toxin is metabolized into less harmful substances by the action of a sophisticated three step detoxification system [6, 7]. In the first step, cytochrome P450 monooxygenases (P450s) act on the toxic compounds to decrease their toxicity. The products of these reactions subsequently become substrates for phase two enzymes, such as glutathione-S-transferases (GSTs) and UDP-glucuronosyltransferases (UGTs), which add large, charged side groups onto substrate molecules making them easier to excrete. Finally, membrane-bound ATP-binding cassette (ABC) transporters remove conjugated products from the cell. The Tobacco hornworm (*Manduca sexta*) is a facultative tobacco specialist, and detoxifies ingested nicotine by inducing P450 enzymes [8, 9]. In addition, as a result of a series of naturally-occurring gene duplication events and transposable element (TE) insertions, overexpression of the P450 *Cyp6g1* is primarily responsible for resistance to the insecticide DDT (dichlorodiphenyltrichloroethane) in *Drosophila* [10–12].

There are tens to hundreds of P450s, GSTs, UGTs, and ABC-transporters in eukaryotic genomes, and for most xenobiotics the precise series of enzymes responsible for their *in vivo* metabolism is unknown. We know there is substantial interindividual variation in the response to toxic compounds in animals such as *Drosophila* (e.g., [13–15]) and in the response to drugs in humans (e.g., [16–18]), but we rarely know which genes in the detoxification cascade segregate for functional variation, or understand the mechanisms by which genetic variation at these loci influences phenotype.

Here we characterize naturally-occurring alleles that contribute to resistance to caffeine in *Drosophila*. Caffeine is one of the most widely used psychoactive drugs in humans, acting as a stimulant, and has been employed as a convenient model xenobiotic in *Drosophila* research, where it is known to induce the expression of a number of P450 and GST genes in *Drosophila* S2 cells, larvae and adults [19–22]. However, simply because a gene responds to caffeine challenge by increasing its expression does not necessarily imply the gene segregates for alleles that give rise to variable drug response.

A powerful method to identify natural alleles that contribute to variable xenobiotic response is to employ a large, stable set of highly-recombinant genotypes derived from a multiparental mapping population [23–29]. The *Drosophila* Synthetic Population Resource (DSPR [30, 31]) consists of a set of >1,700 genotyped RILs (Recombinant Inbred Lines) derived from a pair of highly-recombinant synthetic populations each founded by eight strains. Our previous work has demonstrated the high statistical power and excellent mapping resolution of this community resource [30], and we have succeeded in resolving strong candidate genes contributing to nicotine resistance [15], and the response to chemotherapeutic drugs [14, 32].

In this study we identify several QTL contributing to variation in *Drosophila* adult female lifespan during continuous exposure to 1% caffeine, a measure of caffeine resistance. Of the ten mapped QTL, five harbor strong *a priori* candidate detoxification genes (e.g., P450s). One of these genes—*Cyp12d1*—is implicated by our largest-effect QTL, and segregates for copy number variation (CNV) that strongly correlates with phenotype in the DSPR. Statistically accounting for the effect of this variant in a genomewide scan eliminates the QTL harboring the locus. In addition, *Cyp12d1* shows expression induction in response to caffeine, and RNAi knockdown of the gene significantly reduces resistance. A follow-up association scan using the *Drosophila* Genetic Reference Panel (DGRP [33, 34]) reveals no significant associations after genomewide or QTL-specific multiple testing correction. Although not significant, the intermediate-frequency CNV at *Cyp12d1* shows a trend in the expected direction in the DGRP; lines harboring the duplication have marginally higher resistance on average (*p* = 0.065). If the effect of the CNV is modest, and particularly if additional functional alleles contribute to the QTL implicating *Cyp12d1*, the functional loci may be difficult to individually identify by association in a small sample of natural chromosomes.

## Results

### Phenotypic Variation and Heritability

We measured the lifespan of female flies on 1% caffeine-supplemented media for 1,714 RILs from the DSPR, and 165 inbred lines from the DGRP, testing 16.5 females per genotype on average. There is substantial variation in phenotype in both mapping populations (S1 and S2 Figs). The average RIL mean phenotype in the pA DSPR panel is 37.3 hours (SD = 17.82), and in the pB panel is 35.5 hours (SD = 16.87). The average line mean phenotype in the DGRP is higher at 59.8 hours (SD = 20.80), although the range of the genotype means in the two mapping populations is similar (DSPR range = 6.8–119.9 hours, DGRP range = 10.9–112.7 hours). The broad-sense heritability of our measure of caffeine resistance is just over 50% in all populations (DSPR pA and pB = 0.53, DGRP = 0.51). Because we employed line means for mapping, thus reducing environmental variance, the broad-sense heritability of the mean measure of caffeine resistance is around 94% (DSPR pA and pB = 0.95, DGRP = 0.94).

We note that the contribution of variation in lifespan under control, drug-free conditions likely plays a minimal role in our measure of caffeine resistance. Ivanov *et al*. [35] measured the lifespan of virgin females from the DGRP, and for the 155 lines common to both studies there is no statistically-significant correlation between lifespan and caffeine resistance line means (Pearson's *r* = 0.09, *p* = 0.27).

### Mapping Caffeine Resistance QTL in the DSPR

**Fig 1:**
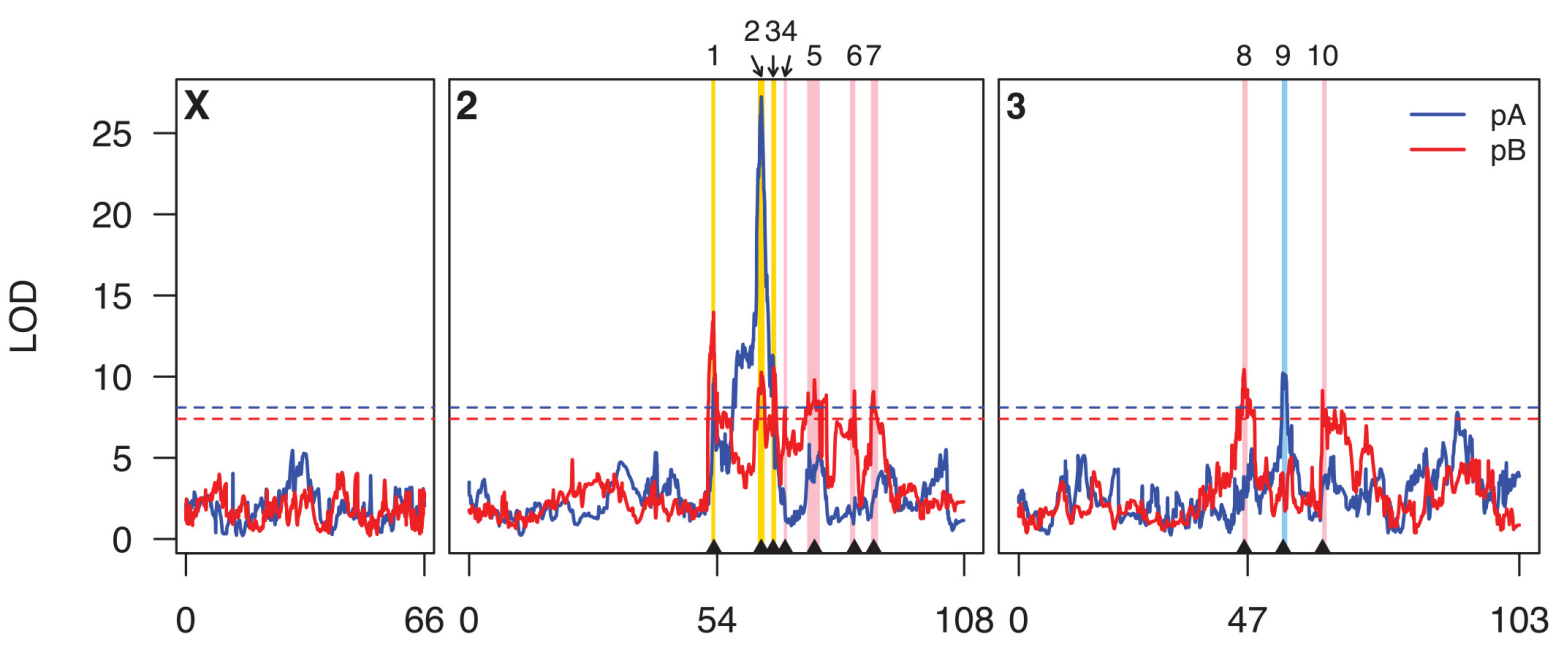
Genome scan for caffeine resistance QTL. Scans for population pA and pB are shown in blue and red curves, respectively, and the horizontal dotted lines represent population-specific genomewide 5% permutation thresholds (pA, LOD = 8.1; pB, LOD = 7.4). Genetic distances along the chromosomes are indicated along the *x*-axis. The centromeres are at positions 54 and 47 on chromosomes 2 and 3, respectively. The positions of the ten QTL we describe in the text are indicated on the plot for ease of reference. Intervals implicated by these QTL are highlighted as vertical bars, with pA-specific QTL in light blue, pB-specific QTL in pink, and QTL identified in both panels in yellow.

Using the same approach we have used previously [15, 31], we mapped QTL for caffeine resistance separately in both pA and pB DSPR mapping populations, identifying ten QTL contributing to resistance (Fig 1 and Tables 1 and S1). Three QTL are common to both populations (i.e., the 2-LOD support intervals overlap), one is unique to pA, and six are unique to pB. Given that the seven unique QTL generally explain smaller fractions of the heritability than the three common QTL (Table 1), it is possible we simply had insufficient power to detect them in the other panel of RILs. However, we cannot discount the possibility that alleles underlying these panel-specific QTL are private to a given panel. Under the assumption the QTL mapped are independent, and act additively, the total variance explained by all mapped QTL is simply the sum of the individual estimated heritability values. On this basis, in the pA population the four mapped QTL explain 31.9% of the broad-sense heritability for the trait, while in pB the nine mapped QTL explain 50.2% of the heritability.

**Table 1:** Details of mapped caffeine resistance QTL.

Name (Chr)	Panel	LOD score	Peak cM (2-LOD CI)^a^	Peak Mb (2-LOD CI)^a^	Number of genes^b^	Percent of *H* ^2^ ^c^
Q1 (*2L*)	pA	9.9	53.3 (53.1–53.6)	18.74 (18.22–19.26)	123	5.5
	pB	14.0	53.2 (52.8–53.3)	18.52 (17.65–18.72)	81	7.7
Q2 (*2R*)	pA	27.2	63.6 (63.3–63.8)	7.07 (6.90–7.16)	36	14.4
	pB	10.3	63.6 (62.9–64.3)	7.06 (6.68–7.43)	122	5.7
Q3 (*2R*)	pA	11.3	66.1 (65.8–66.3)	8.36 (8.21–8.49)	65	6.3
	pB	10.6	66.3 (66.2–66.8)	8.49 (8.43–8.74)	48	5.9
Q4 (*2R*)	pB	8.1	68.8 (68.5–69.2)	9.65 (9.54–9.81)	26	4.5
Q5 (*2R*)	pB	9.8	75.2 (73.6–76.3)	11.49 (11.11–11.76)	68	5.4
Q6 (*2R*)	pB	9.1	83.9 (82.9–84.1)	13.77 (13.46–13.83)	69	5.1
Q7 (*2R*)	pB	9.1	88.1 (87.5–89.0)	15.23 (15.02–15.58)	74	5.0
Q8 (*3L*)	pB	10.4	46.4 (46.0–47.1)	20.56 (19.89–24.36)	398	5.8
Q9 (*3R*)	pA	10.2	54.4 (54.1–55.2)	9.87 (9.73–10.41)	79	5.7
Q10 (*3R*)	pB	9.1	62.5 (62.4–63.4)	14.01 (13.98–14.36)	61	5.1

^a^ 2-LOD CI indicates the 2-LOD support interval of the QTL. Physical positions are given based on release 5 of the *Drosophila* reference genome.

^b^ The number of protein-coding genes in the 2-LOD support interval.

^c^ The proportion of the phenotypic variance due to each QTL comes directly from the linear model used for mapping (page 77 of [76]). The percentage of the broad-sense heritability (*H* ^2^) is simply this estimate divided by the broad-sense heritability of the mean measure of caffeine resistance.

Eight of the ten QTL map away from centromeres (Fig 1), and are mapped to relatively short intervals of 260–750 Kb that include modest numbers of protein-coding genes (mean = 64.8, range = 26–122; S2 Table). Q1 and Q8 are close to the chromosome 2 and chromosome 3 centromeres, respectively, and map to much broader genomic intervals, suggesting it may be more difficult to resolve the underlying causative loci. In terms of *a priori* candidate genes—genes one might anticipate being involved in the differential metabolism of xenobiotics—four QTL intervals contain one or more P450 genes (Q1, Q2, Q3, and Q9), one contains a carboxylesterase (Q8), and one contains two ABC transporters (Q1, although these two genes are only within the Q1 interval implicated in pA, not also the interval implicated in pB). None contain GSTs or UGTs.

If the locus responsible for each of the three shared QTL (Q1, Q2, and Q3) is the same in both panels, the causative gene should be present at a position implicated by *both* the pA and pB QTL LOD support intervals. For Q1 the area implicated by both mapped intervals is 500 kb, about half the width of the panel-specific intervals (Table 1), and harbors just 48 protein-coding genes. For Q2 the area implicated by both QTL is the same as that implicated by pA alone, containing 36 genes (Table 1), and for Q3 the area implicated by both QTL is just 60 Kb and contains ten genes. Each of these three small sets of genes suggested by the intersection of the pA and pB QTL support intervals contains a P450 gene (Q1 harbors *Cyp310a1*, Q2 harbors *Cyp12d1*, and Q3 harbors *Cyp301a1*). These are strong *a priori* candidates to contribute to variation in resistance to xenobiotics.

**Fig 2:**
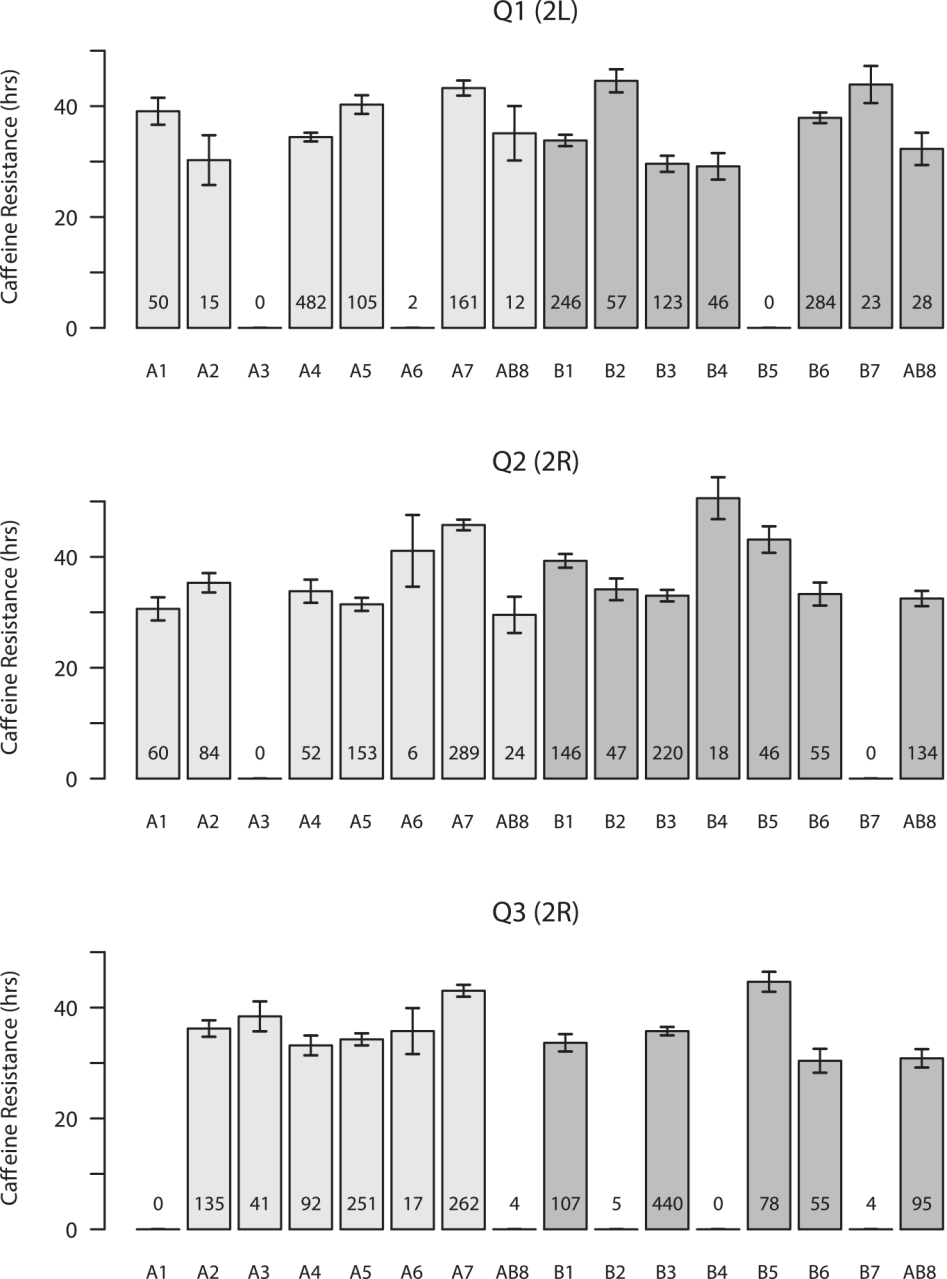
Founder haplotype means and 1-SDs for the three QTL mapped in both panels of DSPR RILs. Means are presented for both pA (light gray bars) and pB (dark gray bars), and the number of RILs for which we assign a founder genotype (probability > 0.95) is listed at the bottom of each bar. Only founder means associated with at least 5 observations are presented. Since line AB8 was used to found both synthetic populations, and the haplotype mean was independently estimated from pA and pB RILs, the line is presented twice in each plot.

For each QTL we estimated the phenotypic effects associated with each founder haplotype (Figs 2 and S3). In many cases it is difficult to clearly discriminate a pattern indicating that just two functional alleles, i.e., "high" and "low" resistance alleles, segregate at mapped loci. It is possible these QTL represent loci segregating for a series of alleles, as we have suggested previously for toxicity and expression QTL mapped in the DSPR [32, 36], and others have observed for QTL mapped in other populations derived from multiple founders [26, 37]. Nevertheless, given we are mapping QTL to intervals ~500-kb, and because multiple very-closely linked QTL would also be expected to yield non-biallelic patterns of founder effects, we cannot be confident that QTL are truly multi-allelic.

Copy number variants (CNVs) are strong candidates to contribute to phenotypic variation [38–40]. One of the best described examples of a functional CNV is at the P450 gene *Cyp6g1* in *D*. *melanogaster*, where a series of structural alleles confer varying levels of resistance to the pesticide DDT [10–12]. Previous work has observed variation in copy number at the *Cyp12d1* locus—under our QTL Q2—in natural populations [41–43], with the *D*. *melanogaster* reference strain possessing two copies with nearly identical gene sequences, *Cyp12d1-d* and *Cyp12d1-p* [44, 45]. Using PCR assays we found that DSPR founders A7, B1, B5, and B7 also possess these two gene copies, while the other 11 founders are single copy. Founder haplotypes at Q2 that harbor two copies of *Cyp12d1* often have higher caffeine resistance than single-copy founders (Fig 2), although the founder with the highest mean resistance (B4) only harbors one copy of *Cyp12d1*. Considering individual RIL phenotypes, in both populations RILs with two copies of *Cyp12d1* survive significantly longer on caffeine than RILs with only one copy of this gene (Welch's *t*-test, *p* < 0.0001 in each population; S4 Fig). To explore whether the CNV can account for the mapped QTL Q2 we rescanned the genome after statistically controlling for the CNV status of each RIL. While most other QTL are still present, after adjusting for the effect of the *Cyp12d1* CNV the signal at Q2 is entirely removed from both DSPR panels (S1 Table and S5 Fig). Thus, it is likely that the CNV at *Cyp12d1*, and/or a nearby variant or variants in strong LD with this event, plays a role in variable caffeine resistance in the DSPR.

### Expression Candidate Genes

Previous work on the genetic basis of xenobiotic resistance has made extensive use of genomewide expression studies to investigate both the innate differences between strains susceptible and resistant to a given compound, and any changes in gene expression that occur in an organism following exposure to the toxic substance. We took pA RILs with either very high or very low caffeine resistance, generated adult females heterozygous between lines of similar phenotype, and exposed groups of these flies both caffeine-supplemented and control media for short periods. Using heterozygous animals minimizes inbreeding effects that may contribute to gene expression, but does make the assumption that heterozygous progeny are phenotypically similar to their parents. Following RNA extraction we pooled samples within phenotypic class (high or low resistance) and treatment, and carried out RNAseq, generating genomewide expression measures for four conditions, and leading to four contrasts of interest (Low-control *versus* High-control, Low-caffeine *versus* High-caffeine, Low-control *versus* Low-caffeine, High-control *versus* High-caffeine). A total of 242 genes were differentially-expressed, i.e., survived a genomewide per-contrast FDR threshold of 5% in at least one contrast (S3 Table). At least 10% of these genes are named members of recognized detoxification families, including P450s (16 genes), GSTs (5 genes), UGT genes (3 genes), and ABC transporters (5 genes).

The first two contrasts allow identification of genes having expression differences between genotypes with different phenotypes; 34 and 37 genes significantly change in expression between Low and High resistance genotypes on control food and caffeine-supplemented food, respectively. Eleven of these genes are shared between contrasts, and for all eleven the direction of the expression change is preserved over contrasts. The second two contrasts allow us to identify genes that change in expression on exposure to caffeine; in the Low (High) resistance genotype 178 (125) genes are differentially-expressed between control and caffeine food. One-hundred and one of these genes are shared between contrasts, and again the direction of the expression changes are consistent between genotypes; caffeine either induces expression in both genotypes or represses expression in both. Willoughby *et al*. [22] previously used a detoxification gene-specific microarray to identify 16 genes (11 P450 genes, 5 GSTs) upregulated in *D*. *melanogaster* third-instar larvae on exposure to caffeine. Ten of these 16 are induced in response to caffeine in both High and Low DSPR resistance genotypes, validating our RNAseq, and suggesting adults and larvae may respond to caffeine challenge via similar mechanisms.

Since our RNAseq study employed pA-derived genotypes, expression changes at genes within QTL intervals mapped in the pA population (Q1, Q2, Q3, and Q9) are of particular interest. Collectively, seven genes under these QTL show a change in expression that survives a genomewide multiple-testing threshold (5% FDR) in at least one experimental contrast (Table 2). Four of these genes are annotated, but un-named genes with limited functional information in FlyBase [45]. One additional gene, *Cpr49Ae*, under Q3 produces a constituent of chitin, and is expressed at higher levels in genotypes that are more resistant to caffeine (Table 2). As a class, cuticle genes were found to be enriched among the set of genes overexpressed in a *D*. *melanogaster* strain resistant to α-amanitin, a toxin produced by a number of species of mushroom [46]. Higher expression of *Cpr48Ae* in caffeine resistant genotypes could plausibly be responsible for providing additional surface protection against xenobiotic exposure. Nonetheless, it is worth noting that *Cpr48Ae* is implicated only by Q3 mapped in pA, and is not present within the interval mapped in pB.

**Table 2:** Differential expression of genes beneath QTL mapped in pA.

		FPKM ^a^				Fold change ^b^			
QTL	Gene	Low (Con)	High (Con)	Low (Caff)	High (Caff)	Low *vs* High (Con)	Low *vs* High (Caff)	Con *vs* Caff (Low)	Con *vs* Caff (High)
Q2 ^c^	*CG13215*	18.8	13.3	4.9	2.5	−0.51	−0.98	−1.94 **	−2.41 **
Q2 ^c^	*Cyp12d1* ^e^	26.3	102.3	293.3	700.8	1.96 **	1.26 **	3.48 **	2.78 **
Q3 ^d^	*CG8834*	80.3	90.5	29.7	38.6	0.17	0.37	−1.43 **	−1.23 **
Q3 ^d^	*CG13160*	1.2	0.7	0.2	0.2	−0.82	0.18	−2.94 **	−1.94 *
Q3 ^d^	*Cpr49Ae*	37.7	77.6	25.1	50.6	1.04 **	1.01 **	−0.58 *	−0.62 *
Q9	*CG33109*	34.6	39.0	16.4	22.3	0.17	0.45	−1.08 **	−0.80 *
Q9	*Cyp6d5*	85.3	175.8	737.3	768.4	1.04	0.06	3.11 **	2.13 **

^a^ Fragments Per Kilobase of transcript per Million mapped reads (values from Cuffdiff output files) for each of the four line/treatment combinations (Con = control food, Caff = caffeine food).

^b^ The log_2_ fold change (second sample divided by first sample) in gene expression for each of the four contrasts.

** indicates the test survives a per-contrast FDR of 5% (i.e., *q* < 0.05).

* indicates the test is significant at *p* < 0.05. No asterisk indicates the test is not significant at a nominal 5% level.

^c^ These genes are also within the Q2 interval mapped in pB.

^d^ These genes are not within the Q3 interval mapped in pB.

^e^ This is the expression measured at the annotated *Cyp12d1-p* gene, with the annotation for the nearly identical gene *Cyp12d1-d* removed from the reference prior to RNAseq analysis. Similar results are seen using the *Cyp12d1-d* annotation, and masking the *Cyp12d1-p* gene.

The strongest expression candidates from this RNAseq screen are P450 genes, *Cyp12d1* under Q2, and *Cyp6d5*, one of the two P450s under Q9 (Table 2). As mentioned previously *Cyp12d1* exists in the *D*. *melanogaster* reference strain as a tandemly-duplicated pair of genes (*Cyp12d1-d* and *Cyp12d1-p*), differing by just 3/1,563 coding sequence nucleotides. Recognizing the difficulty distinguishing reads from each gene, we eliminated the annotation for *Cyp12d1-d* during RNAseq analysis, allowing reads from both gene copies to pile up on *Cyp12d1-p*, and report these values in Table 2. Results are essentially identical regardless of which gene copy is masked for analysis. *Cyp12d1* shows strong, significant expression induction on caffeine exposure in both High and Low resistance genotypes, as has been shown previously [22, 47]. In addition, *Cyp12d1* gene expression is significantly higher for High resistance genotypes under both control and caffeine treatments. We note that at least some of the difference between Low and High genotypes is plausibly explained by the known CNV at this gene; Of the 18 RILs comprising each genotype pool, 17/18 Low genotypes are single copy for *Cyp12d1*, while 14/18 High genotypes have two copies. Array-based female head-specific expression data derived from the progeny of crosses between DSPR RILs [36] confirms a significant effect of the *Cyp12d1* CNV on expression of this gene (S6 Fig). *Cyp12d1* is a clear candidate for the causal locus responsible for Q2.

*Cyp6d5* (Q9) shows highly-significant expression induction following caffeine exposure, but there is no significant difference in expression between Low and High genotypes. The second P450 under Q9 (*Cyp313a1*) shows a reduction in expression in response to caffeine in the Low resistance genotypes at a nominal level (*p* = 0.005), which is not the pattern expected under the assumption that higher expression leads to greater resistance to the drug. Thus, *Cyp6d5* is the stronger candidate to causally underlie Q9. Neither of the P450s under Q1 or Q3 mapped in pA (*Cyp310a1* and *Cyp301a1*, respectively) show significant expression variation across samples even at a nominal, *p* < 0.05 level (S1 Dataset). This result doesn't rule out the possibility the genes contribute to variable resistance to caffeine, but suggests any such role may not involve regulatory variation.

### Functional Validation of P450 Gene Effects on Caffeine Resistance

**Fig 3:**
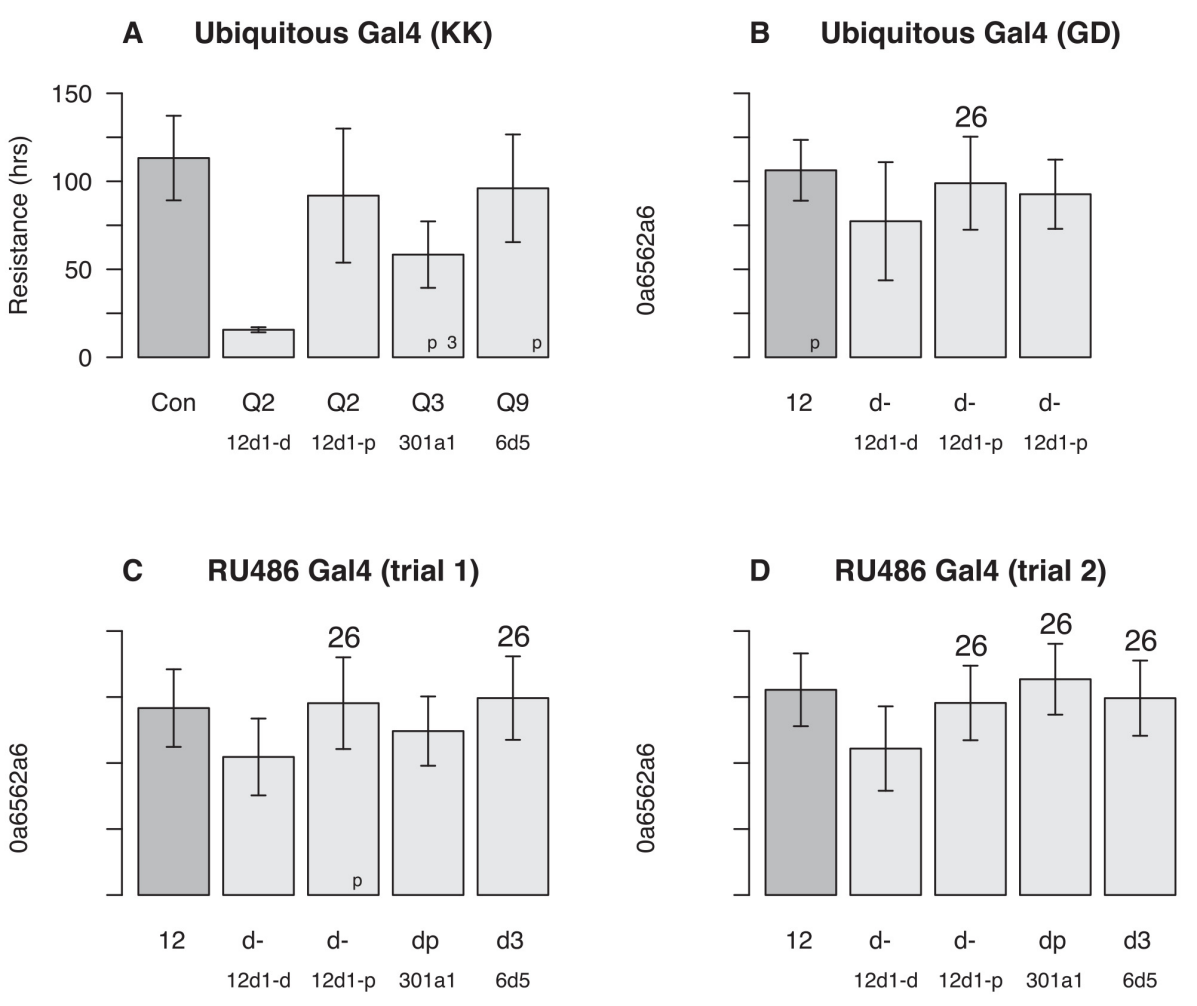
Effects of single gene RNAi knockdown experiments. Gal4-UAS-RNAi female progeny of several genotypes were tested in our caffeine resistance assay against control strains ("Con"). The genes tested were *Cyp12d1-d* and *Cyp12d1-d* under QTL Q2, *Cyp301a1* under Q3, and *Cyp6d5* and under Q9. Each bar represents the mean lifespan (± 1-SD) in the assay across a number of genetically-identical individuals (sample size is in the bottom right corner of each bar), and asterisks represent the significance of Welch's *t*-test comparing each RNAi genotype to its respective control (^ns^ = not significant, *p < 0.01, **p < 0.001, ***p < 10^−10^). (**A**) Driving Gal4 using a ubiquitous promoter with KK-UAS lines. Left-to-right the VDRC stock numbers of the test genotypes are 60100, 109248, 109256, 109771, and 107641. (**B**) Driving Gal4 using a ubiquitous promoter with GD-UAS lines. Left-to-right the VDRC stock numbers of the test genotypes are 60000, 50507, 21235, and 49269. (**C** and **D**) Driving Gal4 ubiquitously in adults using an RU486-inducible promoter in two independent trials, the first (**C**) with flies on RU486 for 24 hours prior to the assay and throughout, and the second (**D**) with flies on RU486 for 48 hours prior to the assay and throughout. Genotypes tested are the same as those depicted in (**A**).

To confirm effects of a subset of the P450 genes uncovered by our mapping and expression studies (*Cyp12d1-d* and *Cyp12d1-p*, *Cyp301a1*, and *Cyp6d5* under Q2, Q3, and Q9, respectively) we carried out knock-down experiments using the Gal4-UAS RNAi system. When knocked down ubiquitously (RNAi in all cells at all timepoints), all but one of the Gal4-UAS showed a significant reduction in caffeine resistance compared to controls (Fig 3A and 3B), with the remaining genotype showing a minor reduction in resistance (Fig 3B; transformant ID 21235, *Cyp12d1-p*, Welch's *t*-test, *p* = 0.066). In two cases the RNAi appears to interfere with some essential developmental process, since we struggled to generate individuals of the appropriate genotypes for assay (Fig 3A; genes *Cyp12d1-d*, transformant ID 109248, and *Cyp301a1*). Since *Cyp301a1* appears to be involved in cuticle development, and reduced expression of the gene results in cuticle malformation [48], this may have played a role in our failure to generate large numbers of progeny. We also cannot rule out any minor developmental abnormalities in the other RNAi genotypes that may have led to reduced adult fitness, and a nonspecific reduction in caffeine resistance. To control for any such pleiotropic effects of the test genes during development, we subsequently used RU486-inducible Gal4 to drive RNAi in all cells in young adults (Fig 3C and 3D). Over two trials (differing only in the amount of time the test animals were fed RU486 prior to caffeine+RU486 exposure), the only RNAi knockdown to show a consistent, highly-significant reduction in caffeine resistance was for *Cyp12d1-d* (Fig 3C and 3D). Since the effect of RU486 on expression knockdown is dose-dependent [49], it is possible higher concentrations of RU486 could have replicated our findings using the ubiquitous Gal4 driver (Fig 3A), and *Cyp301a1* and *Cyp6d5* remain excellent candidates to underlie QTL Q3 and Q9.

Critical to interpreting RNAi results with *Cyp12d1-d* and *Cyp12d1-p* is the fact that these genes appear to be the result of recent duplication [42], and have nearly identical sequences [44, 45]; The genes differ by 3/1,563 coding sequence nucleotides, leading to 3/521 amino acid differences. The hairpin sequences of the UAS-RNAi constructs targeting one member of the gene pair exactly, exhibit zero (transformant ID 50507), one (transformant IDs 21235, 109248, 109256), or two (transformant ID 49269) nucleotide differences from the other member of the gene pair (S2 Dataset). So it is highly likely that in each *Cyp12d1* knockdown experiment we are reducing the expression of *both* genes, albeit perhaps to different degrees.

### Association Mapping Caffeine Resistance Loci in the DGRP

The DSPR has excellent power to identify QTL contributing to trait variation [30], but given the haplotype structure of the DSPR RILs the approach suffers from a lack of resolution in comparison with population-based association mapping. It is possible to carry out local, QTL-centric association scans in the DSPR by imputing SNP genotypes using the estimated mosaic founder haplotype structure of each RIL, but again LD (linkage disequilibrium) is such that large numbers of SNPs tag the exact same haplotype combinations, precluding localization of causative variants [15, 32].

To attempt to identify candidate causative polymorphisms we used the DGRP [33, 34], a sample of fully-resequenced inbred lines derived from a single collection, to carry out association mapping. Following a GWAS (Genomewide Association Study), testing 1.9 million polymorphic events for association with caffeine resistance, the site with the highest association statistic (*p* = 1.7 × 10^−6^) was far from a genomewide Bonferroni significance threshold of *p* ~ 10^−8^ (S4 Table). The lack of a strong association is unsurprising given the likelihood that causative variants explain relatively small fractions of the variance (Table 1), and the low power in a GWAS with < 200 lines to find sites with such effect sizes [50, 51]. To avoid a harsh, genomewide significance threshold we instead restricted our focus to those variants present within QTL intervals mapped in the DSPR. This procedure is more appropriate given we are attempting to validate mapped QTL, rather than attempting *de novo* discovery in the DGRP. Nevertheless, no site reached a QTL-specific Bonferroni threshold, and only one site beneath a mapped QTL reached *p* < 10^−5^ (one of the 24,782 tested variants within the large Q8 interval). Tens to hundreds of variants within QTL intervals have *p*-values that reach a nominal 5% threshold, but in each case the fraction of such variants is essentially what would be expected by chance. We additionally examined associations within 5-kb of the start and end of each of the six annotated P450 genes we have discussed previously (*Cyp12d1-d* and *Cyp12d1-p*, *Cyp301a1*, *Cyp310a1*, *Cyp313a1*, *Cyp6d5*), but again no site in these regions survived a per-gene Bonferroni correction for multiple tests.

In the DSPR we found evidence supporting the role of copy number variation at *Cyp12d1* in conferring resistance to caffeine. Just as for the DSPR we genotyped lines from DGRP using PCR based assays [12, 42], and confirmed our data with bioinformatic calls based sequencing read-depth [41]. Using only those 151 DGRP lines for which we had caffeine phenotypes, where we were confident the lines were homozygous at the *Cyp12d1* locus, and where PCR-based calls and bioinformatics calls were identical, the mean resistance of DGRP lines carrying one *Cyp12d1* copy was 57.6 hours (SD = 20.88, N = 119), and the mean resistance of lines with two copies was 65.4 hours (SD = 20.67, N = 32). While there was a trend in the anticipated direction, the effect of the *Cyp12d1* duplication on caffeine resistance is not significant at the 5% level in the DGRP (Welch's *t*-test, *p* = 0.065; S4 Fig). Given power constraints in the DGRP [50] we may be unable to detect the CNV in this population, particularly if we have overestimated the heritability explained by Q2 (Beavis effects [52, 53]), or if the effect of Q2 in the DSPR is not entirely due to the CNV, and other variants at *Cyp12d1* are additionally causative. However, we cannot rule out a scenario in which the *Cyp12d1* CNV has no effect on caffeine resistance in the DGRP.

## Discussion

In this study we employed a large mapping population, the DSPR, to identify loci segregating for naturally-occurring alleles with variable effects on xenobiotic toxicity in *D*. *melanogaster*. Heritability of resistance to caffeine is around 50% in all mapping panels queried in this study, and we succeeded in mapping a number of QTL that collectively explain between a third and half of the broad-sense heritability. Aside from those QTL mapping to centromeric regions, we map QTL to genomic intervals containing fairly modest numbers of protein-coding genes (26–122), and in those cases where a QTL is mapped to the same location in the pA and pB RIL panels, we can further refine the list of the most likely candidates to those genes implicated by both intervals (i.e., 48, 36 and 10 genes for Q1, Q2, and Q3, respectively). Dissection with this level of resolution is difficult with standard, 2-parental QTL mapping using a single generation of meiotic recombination (i.e., using an F_2_ or backcross mapping design), and can only reasonably be achieved for linkage mapping designs employing populations subjected to multiple rounds of meiotic recombination (e.g., [54]).

Short QTL mapping windows facilitate the identification of true causative gene(s), and in this study our ability to suggest plausible functional loci was additionally enhanced by the nature of our target phenotype. Genes involved in metabolic detoxification pathways represent strong *a priori* candidates to harbor segregating variation contributing to caffeine resistance. Indeed, four caffeine resistance QTL harbor P450 genes (Q1—*Cyp310a1*, Q2—*Cyp12d1*, Q3—*Cyp301a1*, Q9—*Cyp6d5* and *Cyp313a1*), many of which appear to be involved in detoxification [55], and these present excellent targets for future research to identify the precise sequence variants impacting resistance to caffeine. Our mapping results, in combination with RNAseq and RNAi data, imply that a significant fraction of natural variation in resistance to caffeine is due to metabolic detoxification driven by changes in P450 genes (see also [47]).

The strongest candidates from our study are *Cyp12d1* and *Cyp6d5*. These loci are present within QTL intervals (*Cyp12d1* is additionally present within QTL mapped in both pA and pB mapping panels), show expression induction in the presence of caffeine (Table 2), and a reduction in resistance on RNAi gene expression knockdown (Fig 3). In addition, a number of previous studies have shown effects of these loci consistent with a role in xenobiotic metabolism: Both genes are expressed in the larval midgut [56], a likely site of xenobiotic metabolism, both have been shown to be induced by caffeine and phenobarbital [22], and silencing both genes alters downstream metabolism of caffeine [47].

We also show that the copy number variation segregating at *Cyp12d1* [12, 41, 42] associates with our caffeine resistance phenotype in the DSPR RILs. Gene duplications are known to be important contributors to complex variation (e.g., [57]), and evidence from other studies demonstrates that structural variation at P450 genes can be associated with toxin resistance. For instance, an allelic series of CNV events and TE insertions at *Cyp6g1* impacts DDT resistance in *D*. *melanogaster* [12], although in this same study no association was seen between *Cyp12d1* copy number and DDT resistance. In the DGRP, the *Cyp12d1* CNV shows an effect on caffeine resistance in the same direction as in the DSPR, though this trend is not significant at the 5% level (*p* = 0.065). Heterogeneity in the genetic architecture of caffeine resistance across mapping panels could explain the disconnect among studies, with copy number at *Cyp12d1* positively, and causally, associating with resistance in the DSPR but not in the DGRP. Alternatively, the CNV could be in strong LD with the true causative variant in the DSPR, whereas these events may not be in LD in the DGRP. Notably however we did not identify any other associations around *Cyp12d1* in the DGRP that could represent this indirect association. Given the multiple lines of evidence supporting an effect of the *Cyp12d1* locus, it remains highly likely that this gene is involved in caffeine resistance. Future targeted fine-mapping and/or functional genomics work will be required to localize the causative variant(s) in the *Cyp12d1* region that lead to natural variation in caffeine resistance.

Other P450s implicated (*Cyp301a1*, *Cyp310a1*, and *Cyp313a1*) in our mapping did not show changes in expression, either between susceptible or resistant genotypes, or between control and caffeine-supplemented media (S1 Dataset). This may imply that the effect of these genes on variable caffeine resistance is not regulatory in origin, and thus cannot be captured by RNAseq. Alternatively, overall expression levels may be too low—which is likely true for *Cyp301a1* (FPKM = 1.88–2.24) and *Cyp310a1* (FPKM = 0.52–0.67)—and any expression differences may be subtle, precluding the identification of significant expression changes without high levels of biological replication, or by carrying out RNAseq on the likely tissues in which the detoxification process takes place (e.g., [56]). However, without further validation it is not possible to exclude the possibility that these P450 genes are not the causative loci, and instead are merely closely linked to the true caffeine resistance genes.

For the six QTL not containing P450s, all of which have modest effects and were mapped only in the pB panel of DSPR RILs, it is difficult to suggest likely causative genes solely from the lists of implicated loci. QTL Q8 does harbor a carboxylesterase, *Est-Q*, that is known to show expression in the larval midgut, a site of xenobiotic metabolism [56]. However, while this is a plausible candidate gene, because Q8 implicates a total of 398 genes, other evidence for a role in caffeine resistance would be desirable prior to any attempt to functionally validate this gene. Similarly, the *Smc5* gene, shown to be required for resistance to caffeine exposure during development [58] is also present within Q8, and remains a plausible candidate. The genes *cnc*, *Hr96*, and *Keap1* are known to regulate the transcriptional response to xenobiotic challenge in *Drosophila* [20, 59], and *ERR* is associated with the regulation of a number of P450 genes [60]. However, none of these genes are located under the caffeine resistance QTL we map here, suggesting that natural variation in the regulation of the overall transcriptional response to xenobiotics is not strongly involved in caffeine resistance, at least in our assay. Gustatory receptors have been shown to be involved in the avoidance of noxious substances, and *Gr66a* and *Gr93a* are required to avoid caffeine ingestion [61, 62]. Neither of these genes are implicated by our mapped QTL, although other members of the family are present in mapped intervals (*Gr47b* is present within the Q2 interval mapped in pB, and *Gr77a* is present under Q8).

The absence of clear candidate genes underlying mapped QTL is emblematic of the challenge facing most linkage mapping studies. Here, we used a combination of RNAseq and RNAi to validate the effects of certain P450 genes on caffeine resistance, and to refine our list of potential genes for those QTL without obvious a *priori* candidates. Moving from causative genomic regions to the true causative sites is an ongoing challenge for mapping studies. Combining multiple sources of data to provide several lines of evidence supporting candidate loci may prove generally valuable to resolve functional loci within mapped genomic windows, and data on the positions of QTL from this study represent the starting point for such exploration (Tables 1 and S2).

A goal of our research is not only to identify the causative genes, but also to identify the causative sequence variants, and we employed the DGRP association mapping panel to this end [33, 34]. Using a similar phenotyping strategy to that employed in the DSPR, and a straightforward GWAS analysis [33], we found no genomewide significant associations after controlling for multiple tests. This result is likely explained by the low power of the DGRP on a genomewide scale: With 158 lines and a genomewide significance threshold of *p* < 10^−8^, the power to find an intermediate-frequency causative polymorphism (minor allele frequency, MAF = 0.4) that explains 10% of the broad-sense heritability is just 3% (see [50] for description of power calculation).

Due to the lack of power for *de novo* identification of associations in a small GWAS panel, we instead attempted to validate associations within intervals implicated by DSPR QTL, and find associations directly at the six P450 genes suggested by our study. However, even with a lower significance threshold (approaching *p* < 2 × 10^−4^ for the gene-centric association scans) and a concomitant increase in power (62% for a site at a frequency of 0.4 that explains 10% of the variation among lines), we found no QTL-specific variants, and no variants at the set of P450 genes that survive multiple test correction. Thus, power deficits in the DGRP may not entirely explain the lack of overlap between findings in the DSPR and DGRP.

A lack of overlap between the DSPR and DGRP might be expected under a situation in which we have overestimated the heritability explained by QTL mapped in the DSPR. If true QTL effects are much smaller than we estimate (Table 1), power to validate such effects is in turn reduced in the DGRP. Beavis [52] showed that the effects of mapped QTL tended to be overestimated, with the greatest overestimation associated with experiments employing small numbers of samples. Given that the sample size for each DSPR panel was >800 genotypes, and our power to map QTL contributing 5% to variation is over 80% [30], we anticipate that Beavis effects will not be large (see [52] and Figure 15.8 in [63]). Nonetheless, work on the Beavis effect [52, 53] has focused on two-way crosses involving a single-generation of meiotic recombination, and there has been no exploration of the expected magnitude of the Beavis effect in highly-recombinant, multiparental mapping populations.

One possible reason for the failure to find associations in the DGRP within mapped QTL intervals could be that causative variants present in the DSPR are absent in the DGRP. Variants at intermediate frequency will typically be shared by both panels, and given sufficient power, should replicate. Rare variants will be shared less often, and will only replicate some of the time. Those SNPs present in two or more DSPR founders are found in the DGRP >91% of the time, while those SNPs private to a single DSPR founder are seen in the DGRP ~70% of the time (see DSPR founder SNP data from FlyRILs.org and DGRP variant data from dgrp2.gnets.ncsu.edu). In addition, founder means at QTL do not show a pattern consistent with a single founder having a private allele (Figs 2 and S2). Thus, there is limited evidence that the variants responsible for our QTL are unique to the DSPR.

A related possibility is that causative variants have very different frequencies in the DSPR and DGRP. Population-based association mapping approaches struggle to identify low frequency causative variants [50, 64, 65]. Whereas provided the minor allele is captured among one of the founders, a multiparental mapping population has good power to find a low frequency causative variant [30]. Thus, DSPR QTL generated by rare causative loci may not replicate in the DGRP. One piece of evidence against this possibility is the observation that Q1, Q2, and Q3 were all mapped in both DSPR panels. This implies these QTL are not due to rare causative variants present solely in one panel of the DSPR, and suggests the causative variants underlying these QTL are likely present at intermediate-frequency in the DGRP. For instance, the frequency of the duplication allele at the plausible causative CNV at the *Cyp12d1* gene within Q2 is 12.5% among DSPR pA founders, 37.5% among pB founders, and 21.2% among the DGRP lines. Since we do not implicate a causative polymorphism for all other QTL we map in the DSPR it is not possible to determine whether the (unknown) polymorphisms responsible have similar frequencies in the DGRP.

A final possibility for our failure to replicate our QTL mapping results in the DGRP is suggested by the observation from a number of different multiparental mapping studies that QTL frequently segregate for multiple alleles [26, 36, 37]. Similarly, the strain effects for some of the QTL we map here do not show a clear pattern indicative of biallelic QTL simply segregating for a single "high" and "low" allele (Figs 2 and S3). A multiparental panel tests the effect of a local haplotype on phenotype, integrating over any causative variants in a short genomic window. So if QTL mapped in the DSPR are routinely generated by the combined action of multiple variants, each with very small effects, the power to identify any of these individually in the DGRP will be extremely limited. Detection of very subtle effect, intermediate-frequency causative variants, contributing <5% to the natural variation will require massive sample sizes as is common in the human genetics community (e.g., [66]). If instead the multiple causative variants present at genes contributing to polygenic trait variation are all rare, as is the case for genes leading to single-gene, Mendelian disorders, typical GWAS analytical approaches are very poorly powered to detect such genes [65].

In summary, we have used the DSPR to map QTL that collectively explain a large fraction of the natural variation for caffeine resistance in flies. Four of these QTL map to genomic regions containing members of the large family of cytochrome P450 detoxification enzymes. These loci present excellent candidates to harbor variant(s) contributing to phenotypic variation, and for three of these loci—*Cyp6d5*, *Cyp12d1*, and *Cyp301a1*—we validate their effects on caffeine resistance via RNAseq and/or RNAi, suggesting an important role for variation in metabolic detoxification in the control of caffeine resistance. In addition, our data suggests that the number of copies of *Cyp12d1* is a strong determinant of caffeine resistance. We were unable to resolve any additional candidate causative sequence changes by association in a small panel of natural chromosomes, likely due in part to concerns related to power, and potentially as a result of our QTL being generated by the combined action of multiple, small-effect causative alleles. Nonetheless, within each interval implicated by a QTL, tens to hundreds of variants show associations at nominally-significant levels (e.g., *p* < 0.05). If some of these modest-effect associations could be validated in an independent, large association mapping panel this would provide strong evidence for their causative role in affecting phenotypic variation. Ultimately, for strong candidate regions, in the future it will be possible to employ CRISPR-Cas9 genome editing to directly compare alleles at candidate loci in an otherwise identical genetic background to localize regions, and ultimately nucleotide variants directly contributing to phenotypic variance.

## Materials and Methods

### Mapping Populations

To map QTL for caffeine resistance we used RILs from the DSPR (FlyRILs.org). Full details of the mapping panel, its development, the analytical methods employed to map QTL, and simulations supporting the power and resolution of the approach have been described previously [30, 31]. Briefly, the DSPR consists of two panels of >800 RILs (pA and pB). Each set is descended from an advanced generation intercross among eight highly-inbred founder lines, seven of which are specific to a single panel (A1-A7 or B1-B7), with one is common to both panels (AB8). Each intercross population was maintained as a pair of independent replicate subpopulations (pA1, pA2, pB1, pB2) at large population size for 50 generations, after which RILs were generated via 25 generations of full sibling mating. The 15 founder lines have been resequenced, and the RILs genotyped via Restriction site Associated DNA (RAD) tags. These data, along with a hidden Markov model (HMM), allowed the underlying mosaic haplotype structure of each RIL to be determined at a fine scale.

To carry out the GWAS we used strains from the DGRP, a set of inbred lines descended from gravid females caught at a single collection location (Raleigh, NC, USA), and inbred via 20 generations of full sibling mating. DGRP lines were purchased from the Bloomington *Drosophila* Stock Center (flystocks.bio.indiana.edu/). The lines have been fully resequenced, and molecular variation in the panel has been described [33, 34], along with the computational pipeline used to make variant calls [33, 67].

### Caffeine Resistance Assay

Flies from each genotype were allowed to lay eggs for up to 48 hours on standard cornmeal-yeast-molasses food in regular narrow, polystyrene fly vials, and adults were cleared to maintain a roughly equal density of eggs across experimental vials. Eight days after the start of egg laying, any newly emerged adults were cleared from the experimental vials, and two days later 20–25, likely mated female flies were harvested from each vial under CO_2_ anesthesia. These flies were kept as groups on standard media and allowed to recover for 24 hours prior to caffeine challenge.

Our measure of caffeine resistance for a genotype is taken as mean lifespan on media supplemented with 1% caffeine (C0750, Sigma), the same concentration as employed in a previous study of caffeine resistance variation in *D*. *melanogaster* [13]. To increase throughput, and provide for relatively automated data collection, we made use of the *Drosophila* Activity Monitoring System (DAM2, TriKinetics, Inc.). Twenty-four hours before the start of the assay we made cornmeal-yeast-dextrose media, adding caffeine at ~50°C to minimize any heat-induced degradation. We replaced the molasses from our typical culture medium with dextrose to achieve better batch-to-batch reproducibility since small volumes of molasses proved difficult to dispense accurately. The molten media was poured into 100mm diameter petri dishes and allowed to set for 2–3 hours. We pushed the ends of large bundles of polycarbonate activity monitor tubes (5mm diameter × 65mm length) into the media, filling each tube to a height of ~10mm, cleaned the tubes to remove excess media, and sealed the food plug in each tube with molten paraffin wax. On the day of the assay we aspirated individual, 1–3 day old experimental females into monitor tubes without anesthesia, capped the tubes with small foam plugs cut from Droso-Plugs (Genesee Scientific Corporation), and inserted tubes into monitors. All experimental individuals were reared and assayed at 25°C and 50% relative humidity on a 12 hour light/12 hour dark cycle.

The time when experimental flies were introduced to caffeine food in monitor tubes was recorded using a custom Excel macro (Microsoft Corporation), facilitated by arbitrary barcodes on experimental vials and monitors. Activity data was recorded every minute for six days for each experimental block using the DAMSystem3 data collection software (TriKinetics, Inc.), and any permanent cessation of activity was interpreted to be a result of fly death. The data were filtered to remove animals with very low activity levels from the start of the assay, presumably due to damage during aspiration into monitor tubes, and the time until death on caffeine food for each fly was extracted using a series of custom scripts written in R (www.R-project.org).

The above assay was carried out on 1,714 DSPR RILs (853 pA and 861 pB lines) and 165 DGRP lines across 37 experimental blocks. For 1,623 DSPR and 159 DGRP lines we generated a single experimental vial of flies, and tested 16 females from that vial in a single experimental assay block. For a small minority of lines we independently tested 16 flies in each of two blocks, and the correlation between means calculated for each block is high (Pearson's *r* = 0.89, *p* < 10^−15^), giving us confidence in our phenotypic measurements. Over all mapping panel genotypes we scored an average of 16.5 females per genotype (SD = 3.62, range = 9–32). Raw phenotype data is presented in S5 Table.

### Heritability Estimates

Broad-sense heritability of our measure of caffeine resistance (the time until death of an adult female fly on caffeine-supplemented media) was estimated by calculating the genetic and phenotypic variance components from a linear mixed model using the lme and VarCorr functions in the nlme package in R [15, 68]. The estimated genetic variance component divided by the total variance of line means yielded the heritability of the line means that were used for mapping. Heritabilities were separately calculated for both panels of DSPR RILs and the DGRP.

### QTL Mapping in the DSPR

Identification of QTL in the DSPR has been described previously [30, 31]. In essence, for each region in each RIL an HMM is used to assign a probability that the genotype is one of 36 possible homo- or heterozygous founder genotype combinations. Subsequently we assume that the very small number of heterozygous states we find are intermediate between the respective pair of homozygous states, generating eight additive probabilities per position. We then regress the mean line phenotype on these eight probabilities, analyzing the pA and pB panels separately. We did not include a subpopulation covariate since there was no significant difference between the two pA or pB subpopulations in mean RIL caffeine resistance (pA, Welch's *t* = 1.8, *p* = 0.06; pB, Welch's *t* = 1.9, *p* = 0.06). Following Churchill and Doerge [69] we use 1,000 permutations of the data to find appropriate genomewide significance thresholds for QTL identification. We used 2-LOD support intervals to estimate the true positions of causative loci, which simulations suggest correspond to 93–94% confidence intervals for our experimental design, sample size, and mapped QTL effect sizes [30].

We note that there is a clear visual, positive correlation between line mean phenotypes and within-line variance (S1 Fig). This pattern is eliminated following a square root transformation of the line means. However, such a transformation does not change the genomewide likelihood profiles, or alter the QTL we map, and we only report analysis based on the raw, untransformed dataset here.

To account for any effect of the CNV at *Cyp12d1* we performed a similar genomewide scan for QTL as described above, but additionally included a covariate describing the CNV status of each RIL. For this analysis we only used those RILs for which we could be confident of the CNV status, i.e., where the founder allele at the gene was estimated with at least 95% certainty. This resulted in a reduction in the sample size for mapping from 853 to 803 RILs in pA, and from 861 to 812 RILs in pB.

### Genomewide Association Mapping in the DGRP

Phenotype means for 158 DGRP strains assayed were provided to the Mackay lab's web-based analytical engine (dgrp2.gnets.ncsu.edu) to carry out a GWAS using nearly 2 million common (minor allele frequency > 0.05) SNP and non-SNP variants identified in the panel. Prior to the association tests the line means are adjusted to account for any effect of the *Wolbachia* bacteria that infects around half of the lines, and to account for any effects of five major chromosomal inversions: *In(2L)t*, *In(2R)NS*, *In(3R)P*, *In(3R)K*, and *In(3R)Mo*. After which association tests are carried out for each variant using a linear mixed model accounting for variation in relatedness across the lines. See Huang *et al*. [33] for full details.

### RNAseq

We selected two sets of pA RILs that had either very low (in the bottom 2.3% of lines) or very high (in the top 4.2% of lines) caffeine resistance, and crossed RILs in pairs within each phenotypic class. From each cross, we collected 2–3 day old adult female *trans*-heterozygous progeny under CO_2_ anesthesia, allowed the flies to recover for 24 hours, then exposed flies from each genotype either to control food or to 1% caffeine food for 4 hours. No flies died during this short exposure. Flies from each genotype and treatment were then snap-frozen in liquid nitrogen, and total RNA was isolated from each sample using TRIzol reagent (15596–018, Life Technologies). Equal amounts of RNA from each sample were then pooled within each phenotype/treatment combination: susceptible genotypes/control food, susceptible genotypes/caffeine food, resistant genotypes/control food, and resistant genotypes/caffeine food. Ultimately, each of these four pooled samples contained RNA from ~100 experimental females, and haplotypes from 18 pA RILs. The four pooled samples were cleaned through RNeasy Mini columns (74104, Qiagen) following the manufacturer's protocols, used to generate Illumina TruSeq RNAseq libraries, and sequenced over four lanes of an Illumina HiSeq 2500 instrument to generate single-end 50bp reads (Genome Sequencing Facility, KU Medical Center). Raw sequencing reads were deposited in the Sequence Read Archive (SRA) under project accession number SRP051835.

We trimmed raw sequencing reads with sickle (version 1.200, github.com/najoshi/sickle), and used TopHat (version 2.0.9, tophat.cbcb.umd.edu; [70, 71]) to assemble reads from each sample to the *D*. *melanogaster* reference genome (NCBI build 5.3, tophat.cbcb.umd.edu/igenomes.shtml). In order to get an accurate expression measure for the *Cyp12d1* gene, a gene that exists as two, nearly identical copies in the reference (*Cyp12d1-d* and *Cyp12d1-p*) and is subject to copy number variation in the DSPR founders, we modified the reference annotation to eliminate the *Cyp12d1-d* gene. Following TopHat we had 135–169 million quality-trimmed reads per sample, and 89.1–90.4% of these aligned to the reference genome. Subsequently we used Cuffdiff (version 2.1.1, cufflinks.cbcb.umd.edu; [72, 73]) to identify differentially expressed genes (see S1 Text for details of the code and settings). For each comparison between samples we considered only those tests that were successfully executed ('status' column in "gene_exp.diff" Cuffdiff output file has 'OK' flag), and unless otherwise stated only considered genes to be significantly differentially expressed if they survived a Benjamini-Hochberg correction for multiple-testing (*q* < 0.05).

### RNAi

To functionally test four P450 genes implicated in our mapping and RNAseq experiments we employed RNAi using the bipartite Gal4-UAS system, and UAS strains from the Vienna *Drosophila* Resource Center (VDRC [74]). We used strains harboring phiC31-based UAS transgenes, or "KK" lines (107641, *Cyp6d5*; 109248, *Cyp12d1-d*; 109256, *Cyp12d1-p*; 109771, *Cyp301a1*), and strains harboring *P*-element transgenes, or "GD" lines (50507, *Cyp12d1-d*; 21235 and 49269, *Cyp12d1-p*), along with the respective background control lines (60100, KK landing site control strain; 60000, *w* ^*1118*^ host strain for GD library).

It has been reported that the 60100 control strain for the KK library has two landing sites [75]. We used the primers described in Green *et al*. [75] to determine which of the landing sites are occupied in the KK-UAS strains we employed: Strain 109248 has the vector integrated into the annotated target site only, strains 107641 and 109256 have vectors in the newly-identified, previously unannotated site, and 109771 has vectors in both landing sites. These differences in transgene position and number make it more difficult to directly compare effects across genotypes.

We tested the phenotypic effects of RNAi-based expression knockdown using two Gal4 drivers. First, we crossed males from a ubiquitous Gal4 driver under the control of the *Actin 5C* promoter (Bloomington *Drosophila* Stock Center, BDSC number 25374) to females of each UAS or control strain, testing female progeny from these crosses in our standard caffeine resistance assay. Second, we used an RU486-inducible "GeneSwitch" Gal4 driver [49] also under the control of the *Act5C* promoter (BDSC number 9431). Using this strain we again ubiquitously expressed Gal4 in all cells, but exerted some temporal control by driving Gal4 only in young adults. Males from the RU486-inducible Gal4 strain were crossed to females of the KK-UAS and 60100 control strains, and the resulting female progeny fed on media containing RU486 (M8046, Sigma) at 160μg/ml for 24 hours (trial 1) or 48 hours (trial 2) prior to the assay. The resistance assay was conducted as described, except that in addition to 1% caffeine the test media contained 160μg/ml RU486.

### *Cyp12d1* Copy Number PCR

We used primer sets provided in Schmidt *et al*. [12] and McDonnell *et al*. [42] to call copy number variation at the *Cyp12d1-d*/*Cyp12d1-p* locus in lines used in our experiments, and for the DGRP validated our genotype calls against bioinformatic, sequencing read-depth calls from Good *et al*. [41]. CNV genotype calls for the DSPR founders and the DGRP are presented in S3 Dataset, while inferred CNV calls in the DSPR RILs—based on the mosaic haplotype structure of each RIL—are presented in S6 Table.

### Statistical Analysis

All statistics were carried out using core, or custom written, routines using the R statistical programming language (www.R-project.org).

## Supporting Information

S1 Fig Variation in phenotype among DSPR RILs. Means (filled circles) and 1 SDs (vertical lines) for caffeine resistance.
(PDF)

S2 Fig Variation in phenotype among DGRP inbred lines. Means (filled circles) and 1 SDs (vertical lines) for caffeine resistance.
(PDF)

S3 Fig Founder haplotype means and 1-SDs for QTL mapped in just one DSPR population. The number of RILs for which we assign a founder genotype (probability > 0.95) is listed at the bottom of each bar. Only founder means associated with at least 5 observations are presented.
(PDF)

S4 Fig Effect of copy number variation at *Cyp12d1* on caffeine resistance in the DSPR and DGRP mapping panels. The plots show the mean phenotype (± 1-SD) of those lines with one copy of *Cyp12d1* ("single") or two copies ("Dup"), with the number of lines in each class shown at the bottom of each bar. There is a significant effect of the duplication on caffeine resistance in the DSPR pA population (Welch's *t*-test, *p* < 10^−15^) and in the DSPR pB population (Welch's *t*-test, *p* < 10^−5^). However, the effect on the DGRP is not formally significant (Welch's *t*-test, *p* = 0.065).
(PDF)

S5 Fig Genome scan for caffeine resistance QTL after controlling for *Cyp12d1* CNV status. The format of this plot is identical to that of Fig 1 in the main text, but with genomewide 5% permutation thresholds of 8.0 LOD (pA) and 7.2 LOD (pB). In the original genome scan we identified 10 QTL, three in both populations (Q1, Q2, Q3), one in pA only (Q9), and six in pB only (Q4, Q5, Q6, Q7, Q8, Q10). After controlling for the CNV, we continue to identify Q1 in both panels, Q9 in pA only, and Q5, Q6, Q7, Q8, and Q10 in pB only. Q2—the QTL that harbors *Cyp12d1*—disappears in both panels, suggesting much of the effect of that QTL is due to the CNV and/or factors in LD with this variant. Q3 is now only identified in pB, and is absent in pA. The peak at Q4 is also absent. Finally, a peak towards the end of 3R in pA (3R:21960000..22250000), that was just below the significance threshold in our original analysis, is now significant.
(PDF)

S6 Fig Effect of CNV on *Cyp12d1* expression in female heads. King et al. [36] generated array-based, genomewide expression data from female head tissue for 600 genotypes. Each genotype was the result of a cross between an independent pair of DSPR RILs. Using the normalized expression data for *Cyp12d1*, which represents a composite measure of expression from any and all gene copies present in the target genotype since the gene copies are not distinguished on the array, and the CNV status provided in the current study, there is a strong effect of CNV status on *Cyp12d1* expression (linear regression, *p* < 10^−15^).
(PDF)

S1 Dataset Expression levels of the five P450 genes under mapped QTL. Data is taken directly from the "gene_exp.diff" Cuffdiff output file.
(PDF)

S2 Dataset Vienna *Drosophila* Resource Center (VDRC) UAS-RNAi hairpin sequences for *Cyp12d1-d* and *Cyp12d1-p* genes. All data is taken directly from VDRC website (stockcenter.vdrc.at; Accessed January 8, 2015). Hairpin sequences designed to target one member of the gene pair have strong similarity with sequence from the other gene. Bases in hairpin sequences that are different between the two genes are highlighted in green.
(PDF)

S3 Dataset Copy number variation (CNV) genotype calls for the *Cyp12d1* gene region in the DSPR and DGRP mapping panels. (PDF)

S1 Text Code used for RNAseq analysis. (PDF)

S1 Table LOD scores for genomewide QTL scans in the DSPR. The "Chromosome" column indicates the chromosome arm of the position under test (X, 2L, 2R, 3L, and 3R). The "PhysicalPosition" and "GeneticPosition" columns indicate the physical and genetic positions of the site under test, respectively. The "LODscore" columns present the LOD scores at each position in each of the two mapping populations (pA and pB), and in both populations after accounting for *Cyp12d1* CNV status ("CNVadjusted"). The two "VarianceExplained" columns indicate the fraction of the phenotypic variance in the population that is explained by among-line genetic variation at the site under test.
(TXT)

S2 Table Protein-coding genes implicated by mapped QTL. The "QTL" column indicates the QTL interval the gene is within. The "MappingPopulation" column indicates which population (pA and pB) the QTL was mapped in. The "ProteinCodingGene" column gives the symbol for the gene, and "GenePosition" gives the position of the gene in Release 5 of the *Drosophila melanogaster* reference genome.
(TXT)

S3 Table RNAseq-based expression data for all genes surviving a genomewide FDR threshold of 5% in at least one contrast. The data is taken directly from the "gene_exp.diff" Cuffdiff output file, and simply trimmed to remove data for those genes failing to reach genomewide significance (*q* < 0.05) in at least one contrast. The "Chromosome", "GeneStart", and "GeneStop" columns give the position of the gene in the *D*. *melanogaster* reference genome (NCBI build 5.3). The "Sample1" and "Sample2" columns give the names of the two samples being compared, with four contrasts possible: Low-control *versus* High-control, Low-caffeine *versus* High-caffeine, Low-control *versus* Low-caffeine, and High-control *versus* High-caffeine. The "TestStatus" column is derived from Cuffdiff; Only data from tests marked "OK" was used in this study. The columns "Sample1.FPKM" and "Sample2.FPKM" give the FPKM (Fragments Per Kilobase of exon model per Million mapped fragments) values for each sample. The "Log2.FoldChange" column gives the log2 fold change in expression (Sample2 divided by Sample1). The "TestStatistic" column gives the test statistic used by Cuffdiff to compute the significance of the observed change in FPKM between samples, the "Pvalue" column provides the uncorrected *p*-value of the test statistic, and the "Qvalue" column provides the Benjamini-Hochberg FDR adjusted *p*-value of the test statistic.
(TXT)

S4 Table Results of GWAS for caffeine resistance loci using the DGRP. The data is filtered directly from the "gwas.all.assoc" file returned by the Mackay lab DGRP2 analysis pipeline (dgrp2.gnets.ncsu.edu; Accessed June 3, 2014). The "Chromosome" and "Position" columns give information on the variant under test, and the "MAF" column gives the minor allele frequency of the variant in the DGRP. The "Pval" column is the *p*-value of an association test using a mixed-effects model accounting for relatedness among lines. Full details on the analytical pipeline is provided in Huang et al. [33]. Only sites significant at the 1% level (*p* < 0.01) are provided.
(TXT)

S5 Table Raw caffeine resistance phenotypes measured in the DSPR and DGRP mapping panels. The "MappingPanel" column indicates which population the experimental genotype is derived from (the DSPR or DGRP). For DSPR genotypes, columns "Popn" and "PopnRep" indicate which population (pA or pB), and which subpopulation (pA1, pA2, pB1, pB2) the genotype is derived from (for DGRP genotypes all values in these two columns are 'NA'). The "Genotype" column presents the RIL names for DSPR genotypes and the Bloomington *Drosophila* Stock Center codes for the DGRP genotypes. There are multiple rows for any given genotype, with each row containing data for a single female. The "RepVial" column indicates which replicate vial each experimental individual is derived from. In most cases all individuals tested for a genotype come from the same replicate vial, listed as '1'. The "CaffeineResistance" column is the time, in hours, each experimental female survived during continuous exposure to medium supplemented with 1% caffeine.
(TXT)

S6 Table Inferred *Cyp12d1* duplication status for all DSPR RILs. The "RIL" column provides the line code for all 1,715 genotypes phenotyped as part of this study. The "DuplicationStatus" column codes the copy number present at the *Cyp12d1* gene; 0 = single copy, 1 = duplicated, NA = unknown. Only those RILs where the founder genotype at the *Cyp12d1* locus is known with at least 95% certainty are called for copy number variation.
(TXT)

## References

[1] Glendinning JI. How do predators cope with chemically defended foods? Biol Bull. 2007;213(3):252–66. 18083965 10.2307/25066643

[2] Mithofer A, Boland W. Plant defense against herbivores: chemical aspects. Annu Rev Plant Biol. 2012;63:431–50. 10.1146/annurev-arplant-042110-103854 22404468

[3] Zhong W, Maradit-Kremers H, St Sauver JL, Yawn BP, Ebbert JO, Roger VL, et al Age and sex patterns of drug prescribing in a defined American population. Mayo Clin Proc. 2013;88(7):697–707. 10.1016/j.mayocp.2013.04.021 23790544 PMC3754826

[4] Holzinger F, Frick C, Wink M. Molecular basis for the insensitivity of the Monarch (*Danaus plexippus*) to cardiac glycosides. FEBS Lett. 1992;314(3):477–80. 1334851 10.1016/0014-5793(92)81530-y

[5] Zhen Y, Aardema ML, Medina EM, Schumer M, Andolfatto P. Parallel molecular evolution in an herbivore community. Science. 2012;337(6102):1634–7. 23019645 10.1126/science.1226630 PMC3770729

[6] Li X, Schuler MA, Berenbaum MR. Molecular mechanisms of metabolic resistance to synthetic and natural xenobiotics. Annu Rev Entomol. 2007;52:231–53. 16925478 10.1146/annurev.ento.51.110104.151104

[7] Xu C, Li CY, Kong AN. Induction of phase I, II and III drug metabolism/transport by xenobiotics. Arch Pharm Res. 2005;28(3):249–68. 15832810 10.1007/BF02977789

[8] Snyder MJ, Glendinning JI. Causal connection between detoxification enzyme activity and consumption of a toxic plant compound. J Comp Physiol A. 1996;179(2):255–61. 8765561 10.1007/BF00222792

[9] Wink M, Theile V. Alkaloid tolerance in *Manduca sexta* and phylogenetically related sphingids (Lepidoptera: Sphingidae). Chemoecology. 2002;12:29–46.

[10] Chung H, Bogwitz MR, McCart C, Andrianopoulos A, Ffrench-Constant RH, Batterham P, et al *Cis*-regulatory elements in the *Accord* retrotransposon result in tissue-specific expression of the *Drosophila melanogaster* insecticide resistance gene *Cyp6g1* . Genetics. 2007;175(3):1071–7. 17179088 10.1534/genetics.106.066597 PMC1840086

[11] Daborn PJ, Yen JL, Bogwitz MR, Le Goff G, Feil E, Jeffers S, et al A single p450 allele associated with insecticide resistance in *Drosophila* . Science. 2002;297(5590):2253–6. 12351787 10.1126/science.1074170

[12] Schmidt JM, Good RT, Appleton B, Sherrard J, Raymant GC, Bogwitz MR, et al Copy number variation and transposable elements feature in recent, ongoing adaptation at the *Cyp6g1* locus. PLoS Genet. 2010;6(6):e1000998 10.1371/journal.pgen.1000998 20585622 PMC2891717

[13] Carrillo R, Gibson G. Unusual genetic architecture of natural variation affecting drug resistance in *Drosophila melanogaster* . Genet Res. 2002;80(3):205–13. 12688659 10.1017/s0016672302005888

[14] King EG, Kislukhin G, Walters KN, Long AD. Using *Drosophila melanogaster* to identify chemotherapy toxicity genes. Genetics. 2014;198(1):31–43. 10.1534/genetics.114.161968 25236447 PMC4174942

[15] Marriage TN, King EG, Long AD, Macdonald SJ. Fine-mapping nicotine resistance loci in *Drosophila* using a multiparent advanced generation inter-cross population. Genetics. 2014;198(1):45–57. 10.1534/genetics.114.162107 25236448 PMC4174953

[16] Ge D, Fellay J, Thompson AJ, Simon JS, Shianna KV, Urban TJ, et al Genetic variation in *IL28B* predicts hepatitis C treatment-induced viral clearance. Nature. 2009;461(7262):399–401. 10.1038/nature08309 19684573

[17] Shuldiner AR, O'Connell JR, Bliden KP, Gandhi A, Ryan K, Horenstein RB, et al Association of cytochrome P450 2C19 genotype with the antiplatelet effect and clinical efficacy of clopidogrel therapy. JAMA. 2009;302(8):849–57. 10.1001/jama.2009.1232 19706858 PMC3641569

[18] Takeuchi F, McGinnis R, Bourgeois S, Barnes C, Eriksson N, Soranzo N, et al A genome-wide association study confirms *VKORC1*, *CYP2C9*, and *CYP4F2* as principal genetic determinants of warfarin dose. PLoS Genet. 2009;5(3):e1000433 10.1371/journal.pgen.1000433 19300499 PMC2652833

[19] Bhaskara S, Dean ED, Lam V, Ganguly R. Induction of two cytochrome P450 genes, *Cyp6a2* and *Cyp6a8*, of *Drosophila melanogaster* by caffeine in adult flies and in cell culture. Gene. 2006;377:56–64. 16713132 10.1016/j.gene.2006.02.032

[20] Misra JR, Horner MA, Lam G, Thummel CS. Transcriptional regulation of xenobiotic detoxification in *Drosophila* . Genes Dev. 2011;25(17):1796–806. 10.1101/gad.17280911 21896655 PMC3175716

[21] Morra R, Kuruganti S, Lam V, Lucchesi JC, Ganguly R. Functional analysis of the *cis*-acting elements responsible for the induction of the *Cyp6a8* and *Cyp6g1* genes of *Drosophila melanogaster* by DDT, phenobarbital and caffeine. Insect Mol Biol. 2010;19(1):121–30. 10.1111/j.1365-2583.2009.00954.x 20002224

[22] Willoughby L, Chung H, Lumb C, Robin C, Batterham P, Daborn PJ. A comparison of *Drosophila melanogaster* detoxification gene induction responses for six insecticides, caffeine and phenobarbital. Insect Biochem Mol Biol. 2006;36(12):934–42. 17098168 10.1016/j.ibmb.2006.09.004

[23] Churchill GA, Airey DC, Allayee H, Angel JM, Attie AD, Beatty J, et al The Collaborative Cross, a community resource for the genetic analysis of complex traits. Nat Genet. 2004;36(11):1133–7. 15514660 10.1038/ng1104-1133

[24] Kover PX, Valdar W, Trakalo J, Scarcelli N, Ehrenreich IM, Purugganan MD, et al A Multiparent Advanced Generation Inter-Cross to fine-map quantitative traits in Arabidopsis thaliana. PLoS Genet. 2009;5(7):e1000551 10.1371/journal.pgen.1000551 19593375 PMC2700969

[25] Macdonald SJ, Long AD. Joint estimates of quantitative trait locus effect and frequency using synthetic recombinant populations of *Drosophila melanogaster* . Genetics. 2007;176(2):1261–81. 17435224 10.1534/genetics.106.069641 PMC1894589

[26] Rat Genome S, Mapping C, Baud A, Hermsen R, Guryev V, Stridh P, et al Combined sequence-based and genetic mapping analysis of complex traits in outbred rats. Nat Genet. 2013;45(7):767–75. 10.1038/ng.2644 23708188 PMC3821058

[27] Svenson KL, Gatti DM, Valdar W, Welsh CE, Cheng R, Chesler EJ, et al High-resolution genetic mapping using the Mouse Diversity outbred population. Genetics. 2012;190(2):437–47. 10.1534/genetics.111.132597 22345611 PMC3276626

[28] Threadgill DW, Churchill GA. Ten years of the collaborative cross. G3 (Bethesda). 2012;2(2):153–6. 22384393 10.1534/g3.111.001891 PMC3284322

[29] Valdar W, Flint J, Mott R. Simulating the collaborative cross: power of quantitative trait loci detection and mapping resolution in large sets of recombinant inbred strains of mice. Genetics. 2006;172(3):1783–97. 16361245 10.1534/genetics.104.039313 PMC1456308

[30] King EG, Macdonald SJ, Long AD. Properties and power of the *Drosophila* Synthetic Population Resource for the routine dissection of complex traits. Genetics. 2012;191(3):935–49. 10.1534/genetics.112.138537 22505626 PMC3389985

[31] King EG, Merkes CM, McNeil CL, Hoofer SR, Sen S, Broman KW, et al Genetic dissection of a model complex trait using the *Drosophila* Synthetic Population Resource. Genome Res. 2012;22(8):1558–66. 10.1101/gr.134031.111 22496517 PMC3409269

[32] Kislukhin G, King EG, Walters KN, Macdonald SJ, Long AD. The genetic architecture of methotrexate toxicity is similar in *Drosophila melanogaster* and humans. G3 (Bethesda). 2013;3(8):1301–10. 23733889 10.1534/g3.113.006619 PMC3737169

[33] Huang W, Massouras A, Inoue Y, Peiffer J, Ramia M, Tarone AM, et al Natural variation in genome architecture among 205 *Drosophila melanogaster* Genetic Reference Panel lines. Genome Res. 2014;24(7):1193–208. 10.1101/gr.171546.113 24714809 PMC4079974

[34] Mackay TF, Richards S, Stone EA, Barbadilla A, Ayroles JF, Zhu D, et al The *Drosophila melanogaster* Genetic Reference Panel. Nature. 2012;482(7384):173–8. 10.1038/nature10811 22318601 PMC3683990

[35] Ivanov DK, Escott-Price V, Ziehm M, Magwire MM, Mackay TF, Partridge L, et al Longevity GWAS Using the *Drosophila* Genetic Reference Panel. J Gerontol A Biol Sci Med Sci. 2015. 10.1093/gerona/glv047 PMC4631106 25922346

[36] King EG, Sanderson BJ, McNeil CL, Long AD, Macdonald SJ. Genetic dissection of the *Drosophila melanogaster* female head transcriptome reveals widespread allelic heterogeneity. PLoS Genet. 2014;10(5):e1004322 10.1371/journal.pgen.1004322 24810915 PMC4014434

[37] Giraud H, Lehermeier C, Bauer E, Falque M, Segura V, Bauland C, et al Linkage disequilibrium with linkage analysis of multiline crosses reveals different multiallelic QTL for hybrid performance in the flint and dent heterotic groups of maize. Genetics. 2014;198(4):1717–34. 10.1534/genetics.114.169367 25271305 PMC4256782

[38] Stankiewicz P, Lupski JR. Structural variation in the human genome and its role in disease. Annu Rev Med. 2010;61:437–55. 10.1146/annurev-med-100708-204735 20059347

[39] Weischenfeldt J, Symmons O, Spitz F, Korbel JO. Phenotypic impact of genomic structural variation: insights from and for human disease. Nat Rev Genet. 2013;14(2):125–38. 10.1038/nrg3373 23329113

[40] Zichner T, Garfield DA, Rausch T, Stutz AM, Cannavo E, Braun M, et al Impact of genomic structural variation in *Drosophila melanogaster* based on population-scale sequencing. Genome Res. 2013;23(3):568–79. 10.1101/gr.142646.112 23222910 PMC3589545

[41] Good RT, Gramzow L, Battlay P, Sztal T, Batterham P, Robin C. The molecular evolution of cytochrome P450 genes within and between *Drosophila* species. Genome Biol Evol. 2014;6(5):1118–34. 10.1093/gbe/evu083 24751979 PMC4040991

[42] McDonnell CM, King D, Comeron JM, Li H, Sun W, Berenbaum MR, et al Evolutionary toxicogenomics: diversification of the *Cyp12d1* and *Cyp12d3* genes in *Drosophila* species. J Mol Evol. 2012;74(5–6):281–96. 10.1007/s00239-012-9506-3 22811321

[43] Schrider DR, Begun DJ, Hahn MW. Detecting highly differentiated copy-number variants from pooled population sequencing. Pac Symp Biocomput. 2013:344–55. 23424139 PMC3587772

[44] Adams MD, Celniker SE, Holt RA, Evans CA, Gocayne JD, Amanatides PG, et al The genome sequence of *Drosophila melanogaster* . Science. 2000;287(5461):2185–95. 10731132 10.1126/science.287.5461.2185

[45] St Pierre SE, Ponting L, Stefancsik R, McQuilton P, FlyBase C. FlyBase 102—advanced approaches to interrogating FlyBase. Nucleic Acids Res. 2014;42(Database issue):D780–8. 10.1093/nar/gkt1092 24234449 PMC3964969

[46] Mitchell CL, Saul MC, Lei L, Wei H, Werner T. The mechanisms underlying alpha-amanitin resistance in *Drosophila melanogaster*: a microarray analysis. PLoS One. 2014;9(4):e93489 10.1371/journal.pone.0093489 24695618 PMC3973583

[47] Coelho A, Fraichard S, Le Goff G, Faure P, Artur Y, Ferveur JF, et al Cytochrome P450-dependent metabolism of caffeine in *Drosophila melanogaster* . PLoS One. 2015;10(2):e0117328 10.1371/journal.pone.0117328 25671424 PMC4324904

[48] Sztal T, Chung H, Berger S, Currie PD, Batterham P, Daborn PJ. A cytochrome p450 conserved in insects is involved in cuticle formation. PLoS One. 2012;7(5):e36544 10.1371/journal.pone.0036544 22574182 PMC3344891

[49] Osterwalder T, Yoon KS, White BH, Keshishian H. A conditional tissue-specific transgene expression system using inducible GAL4. Proc Natl Acad Sci U S A. 2001;98(22):12596–601. 11675495 10.1073/pnas.221303298 PMC60099

[50] Long AD, Macdonald SJ, King EG. Dissecting complex traits using the *Drosophila* Synthetic Population Resource. Trends Genet. 2014;30(11):488–95. 10.1016/j.tig.2014.07.009 25175100 PMC4253597

[51] Turner TL, Miller PM, Cochrane VA. Combining genome-wide methods to investigate the genetic complexity of courtship song variation in *Drosophila melanogaster* . Mol Biol Evol. 2013;30(9):2113–20. 10.1093/molbev/mst111 23777628 PMC3748354

[52] Beavis W. The power and deceit of QTL experiments: lessons from comparative QTL studies. Proceedings of the 49th Annual Corn and Sorghum Industry Research Conference. Washington, DC: American Seed Trade Association; 1994. p. 250–66.

[53] Xu S. Theoretical basis of the Beavis effect. Genetics. 2003;165(4):2259–68. 14704201 10.1093/genetics/165.4.2259 PMC1462909

[54] Parker CC, Carbonetto P, Sokoloff G, Park YJ, Abney M, Palmer AA. High-resolution genetic mapping of complex traits from a combined analysis of F2 and advanced intercross mice. Genetics. 2014;198(1):103–16. 10.1534/genetics.114.167056 25236452 PMC4174923

[55] Chung H, Sztal T, Pasricha S, Sridhar M, Batterham P, Daborn PJ. Characterization of *Drosophila melanogaster* cytochrome P450 genes. Proc Natl Acad Sci U S A. 2009;106(14):5731–6. 10.1073/pnas.0812141106 19289821 PMC2667016

[56] Harrop TW, Pearce SL, Daborn PJ, Batterham P. Whole-genome expression analysis in the third instar larval midgut of *Drosophila melanogaster* . G3 (Bethesda). 2014;4(11):2197–205. 25193493 10.1534/g3.114.013870 PMC4232545

[57] Stranger BE, Forrest MS, Dunning M, Ingle CE, Beazley C, Thorne N, et al Relative impact of nucleotide and copy number variation on gene expression phenotypes. Science. 2007;315(5813):848–53. 17289997 10.1126/science.1136678 PMC2665772

[58] Li X, Zhuo R, Tiong S, Di Cara F, King-Jones K, Hughes SC, et al The *Smc5*/*Smc6*/*MAGE* complex confers resistance to caffeine and genotoxic stress in *Drosophila melanogaster* . PLoS One. 2013;8(3):e59866 10.1371/journal.pone.0059866 23555814 PMC3610895

[59] King-Jones K, Horner MA, Lam G, Thummel CS. The DHR96 nuclear receptor regulates xenobiotic responses in *Drosophila* . Cell Metab. 2006;4(1):37–48. 16814731 10.1016/j.cmet.2006.06.006

[60] Sun W, Valero MC, Seong KM, Steele LD, Huang IT, Lee CH, et al A glycine insertion in the estrogen-related receptor (ERR) is associated with enhanced expression of three cytochrome P450 genes in transgenic *Drosophila melanogaster* . PLoS One. 2015;10(3):e0118779 10.1371/journal.pone.0118779 25761142 PMC4356566

[61] Lee Y, Moon SJ, Montell C. Multiple gustatory receptors required for the caffeine response in *Drosophila* . Proc Natl Acad Sci U S A. 2009;106(11):4495–500. 10.1073/pnas.0811744106 19246397 PMC2657413

[62] Moon SJ, Kottgen M, Jiao Y, Xu H, Montell C. A taste receptor required for the caffeine response *in vivo* . Curr Biol. 2006;16(18):1812–7. 16979558 10.1016/j.cub.2006.07.024

[63] Lynch M, Walsh B. Genetics and Analysis of Quantitative Traits. Sunderland, Massachusetts: Sinauer Associates, Inc; 1998.

[64] Pritchard JK. Are rare variants responsible for susceptibility to complex diseases? Am J Hum Genet. 2001;69(1):124–37. 11404818 10.1086/321272 PMC1226027

[65] Thornton KR, Foran AJ, Long AD. Properties and modeling of GWAS when complex disease risk is due to non-complementing, deleterious mutations in genes of large effect. PLoS Genet. 2013;9(2):e1003258 10.1371/journal.pgen.1003258 23437004 PMC3578756

[66] DIAbetes Genetics Replication Meta-analysis, et al Genome-wide trans-ancestry meta-analysis provides insight into the genetic architecture of type 2 diabetes susceptibility. Nat Genet. 2014;46(3):234–44. 10.1038/ng.2897 24509480 PMC3969612

[67] Stone EA. Joint genotyping on the fly: identifying variation among a sequenced panel of inbred lines. Genome Res. 2012;22(5):966–74. 10.1101/gr.129122.111 22367192 PMC3337441

[68] Pinheiro J, Bates D, DebRoy S, Sarkar D, Team RDC. nlme: linear and nonlinear mixed effects models. R package version 31–101. 2011.

[69] Churchill GA, Doerge RW. Empirical threshold values for quantitative trait mapping. Genetics. 1994;138(3):963–71. 7851788 10.1093/genetics/138.3.963 PMC1206241

[70] Kim D, Pertea G, Trapnell C, Pimentel H, Kelley R, Salzberg SL. TopHat2: accurate alignment of transcriptomes in the presence of insertions, deletions and gene fusions. Genome Biol. 2013;14(4):R36 10.1186/gb-2013-14-4-r36 23618408 PMC4053844

[71] Trapnell C, Pachter L, Salzberg SL. TopHat: discovering splice junctions with RNA-Seq. Bioinformatics. 2009;25(9):1105–11. 10.1093/bioinformatics/btp120 19289445 PMC2672628

[72] Trapnell C, Hendrickson DG, Sauvageau M, Goff L, Rinn JL, Pachter L. Differential analysis of gene regulation at transcript resolution with RNA-seq. Nat Biotechnol. 2013;31(1):46–53. 10.1038/nbt.2450 23222703 PMC3869392

[73] Trapnell C, Williams BA, Pertea G, Mortazavi A, Kwan G, van Baren MJ, et al Transcript assembly and quantification by RNA-Seq reveals unannotated transcripts and isoform switching during cell differentiation. Nat Biotechnol. 2010;28(5):511–5. 10.1038/nbt.1621 20436464 PMC3146043

[74] Dietzl G, Chen D, Schnorrer F, Su KC, Barinova Y, Fellner M, et al A genome-wide transgenic RNAi library for conditional gene inactivation in *Drosophila* . Nature. 2007;448(7150):151–6. 17625558 10.1038/nature05954

[75] Green EW, Fedele G, Giorgini F, Kyriacou CP. A *Drosophila* RNAi collection is subject to dominant phenotypic effects. Nat Methods. 2014;11(3):222–3. 10.1038/nmeth.2856 24577271

[76] Broman K, Sen S. A Guide to QTL Mapping with R/qtl. New York: Springer Dordrecht; 2009.

