# Correction: Identifying Loci Contributing to Natural Variation in Xenobiotic Resistance in *Drosophila*

**DOI:** 10.1371/journal.pgen.1005830

**Published:** 2016-01-22

**Authors:** The PLOS Genetics Staff

Due to an error in the production process, Fig 3 is incorrect. The authors have provided a corrected version here. The publisher apologizes for the error.

**Fig 3:**
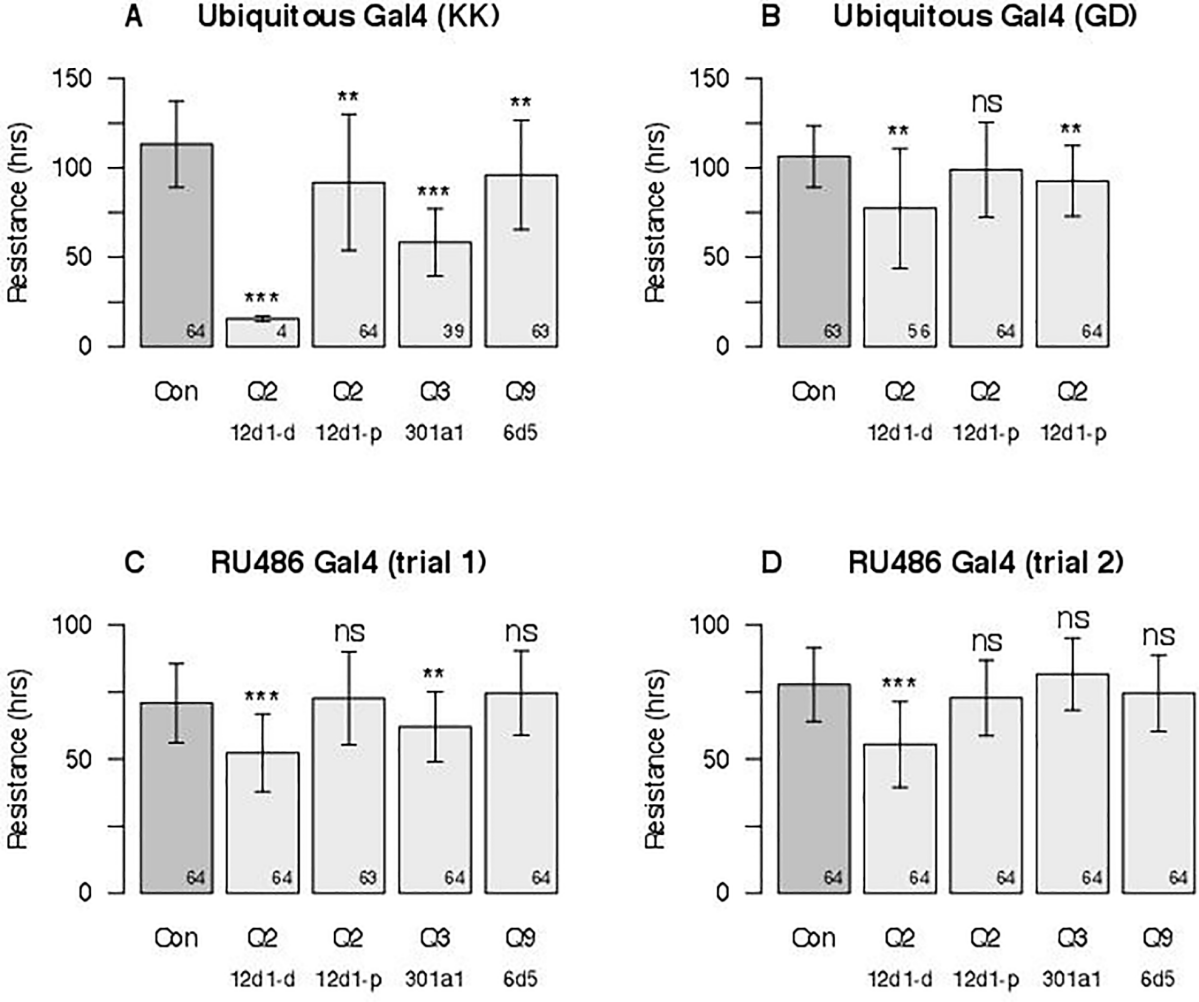
Effects of single gene RNAi knockdown experiments. Gal4-UAS-RNAi female progeny of several genotypes were tested in our caffeine resistance assay against control strains ("Con"). The genes tested were *Cyp12d1-d* and *Cyp12d1-d* under QTL Q2, *Cyp301a1* under Q3, and *Cyp6d5* and under Q9. Each bar represents the mean lifespan (± 1-SD) in the assay across a number of genetically-identical individuals (sample size is in the bottom right corner of each bar), and asterisks represent the significance of Welch's *t*-test comparing each RNAi genotype to its respective control (^ns^ = not significant, *p < 0.01, **p < 0.001, ***p < 10^−10^). (**A**) Driving Gal4 using a ubiquitous promoter with KK-UAS lines. Left-to-right the VDRC stock numbers of the test genotypes are 60100, 109248, 109256, 109771, and 107641. (**B**) Driving Gal4 using a ubiquitous promoter with GD-UAS lines. Left-to-right the VDRC stock numbers of the test genotypes are 60000, 50507, 21235, and 49269. (**C** and **D**) Driving Gal4 ubiquitously in adults using an RU486-inducible promoter in two independent trials, the first (**C**) with flies on RU486 for 24 hours prior to the assay and throughout, and the second (**D**) with flies on RU486 for 48 hours prior to the assay and throughout. Genotypes tested are the same as those depicted in (**A**).
