# Supplementary figures and images for: Identifying Loci Contributing to Natural Variation in Xenobiotic Resistance in Drosophila
Source: PLoS Genet. 2015 Nov 30;11(11):e1005663. doi: 10.1371/journal.pgen.1005663 (PMC4664282; doi:10.1371/journal.pgen.1005663)

## DSPR pA and pB RILs

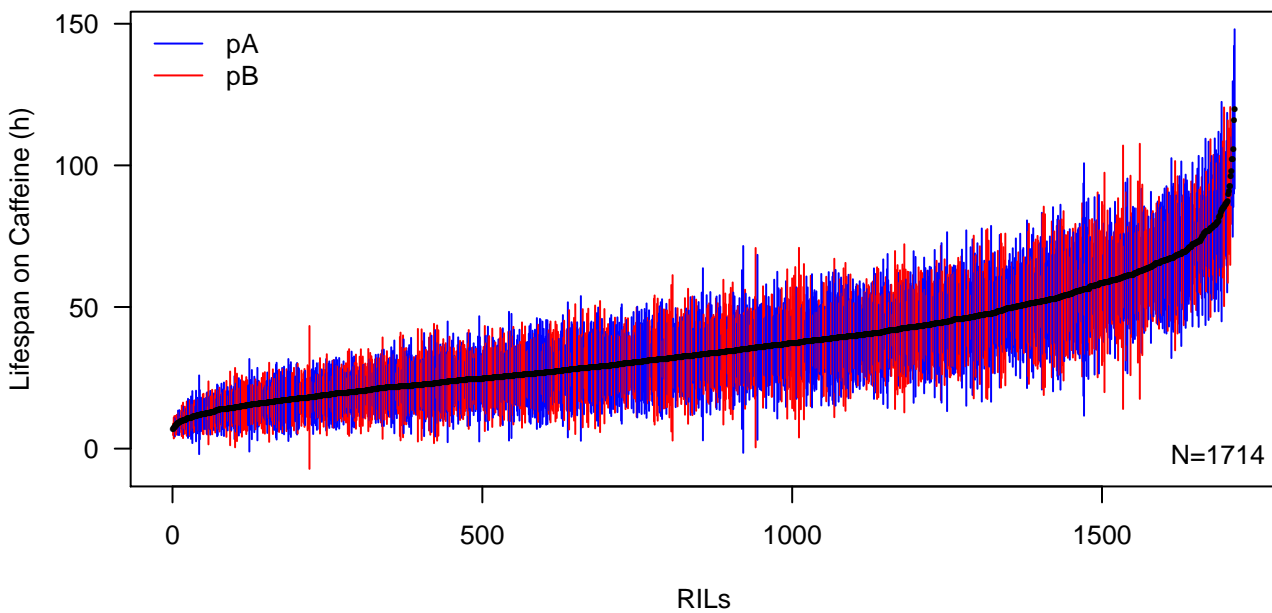

### pA RILs

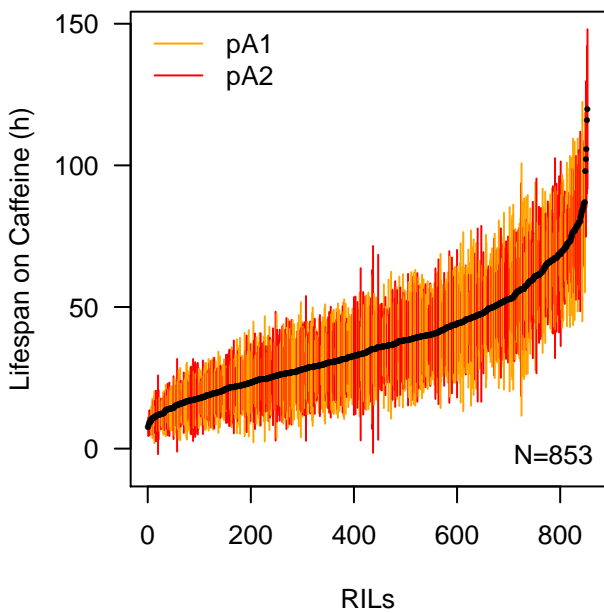

### pB RILs

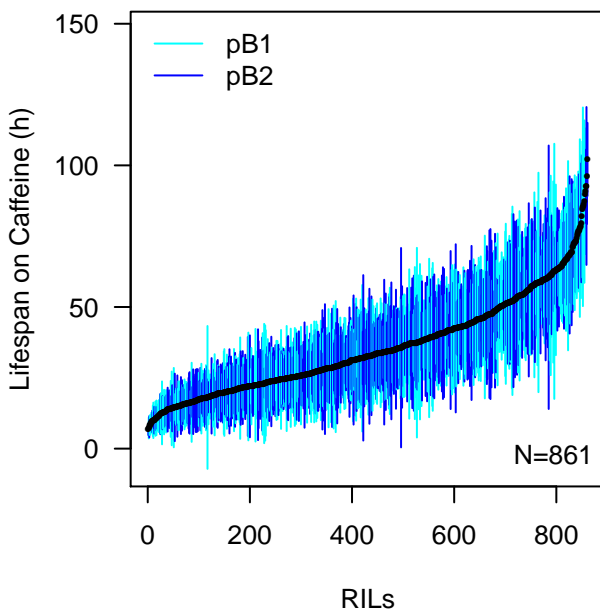

Supplement: S1 Fig — Means (filled circles) and 1 SDs (vertical lines) for caffeine resistance. (PDF) [file pgen.1005663.s001.pdf]

## DGRP Lines

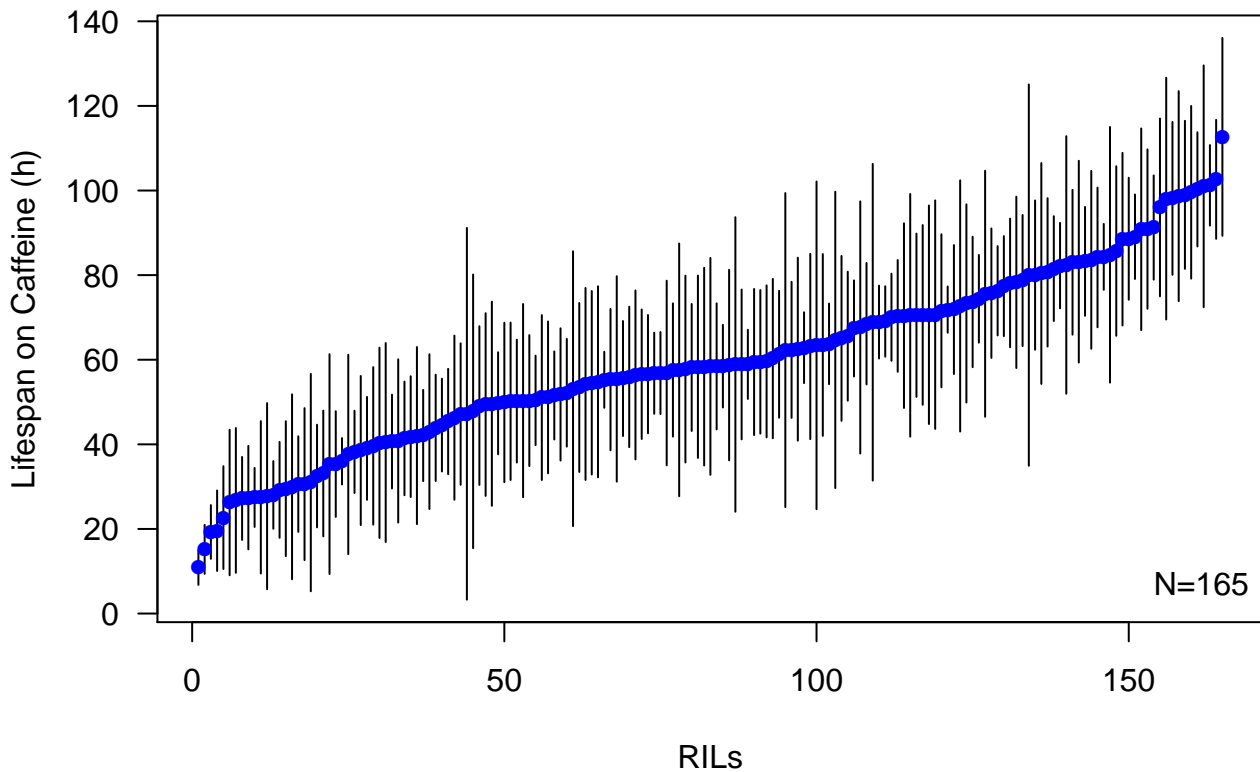

Supplement: S2 Fig — Means (filled circles) and 1 SDs (vertical lines) for caffeine resistance. (PDF) [file pgen.1005663.s002.pdf]

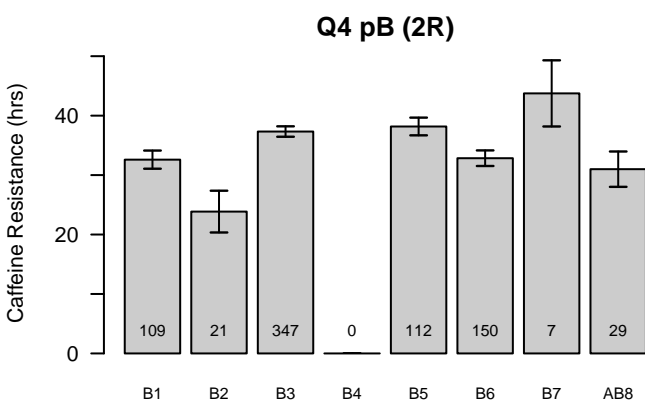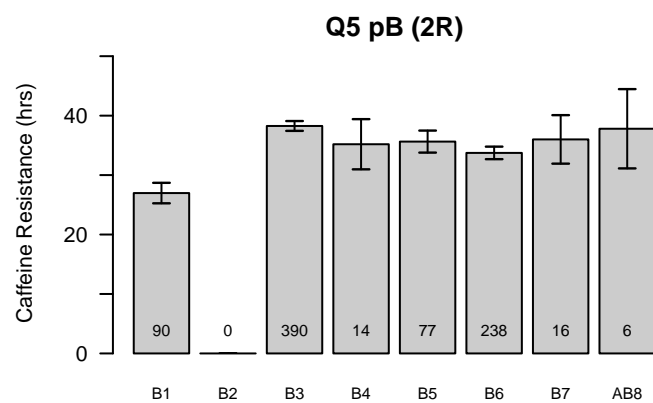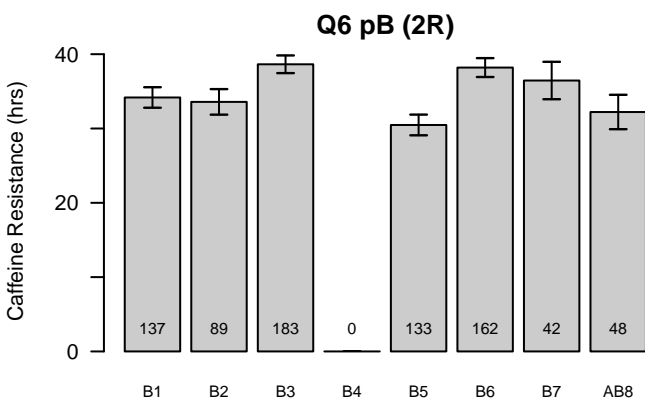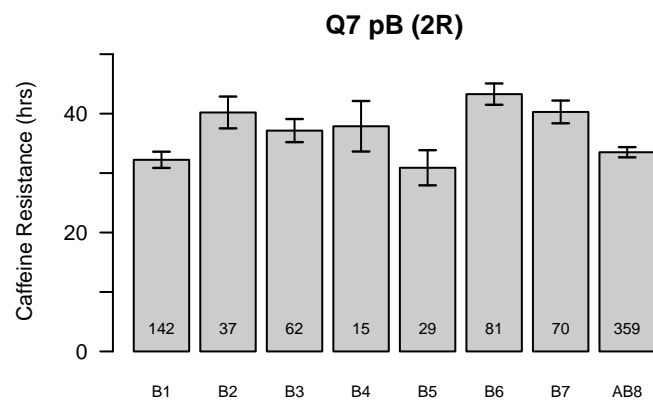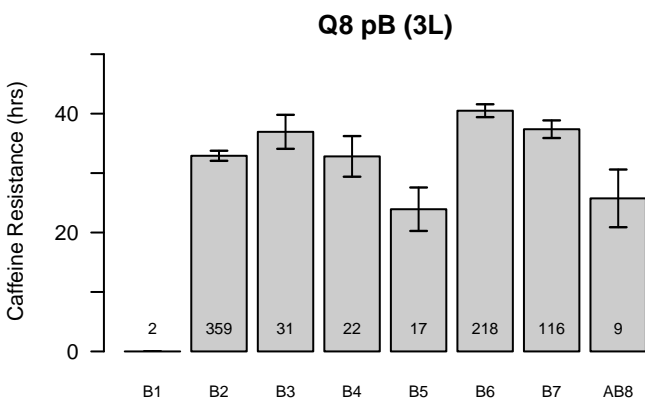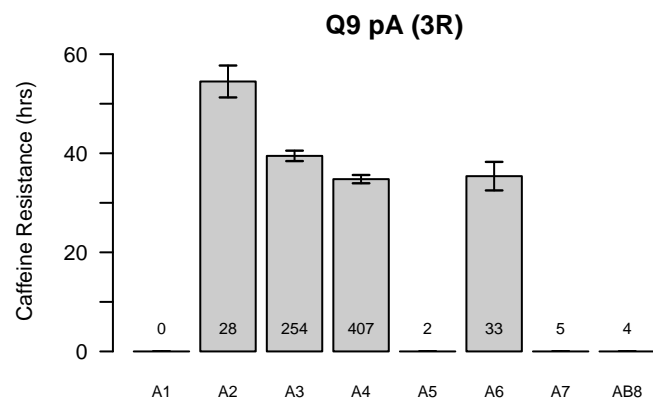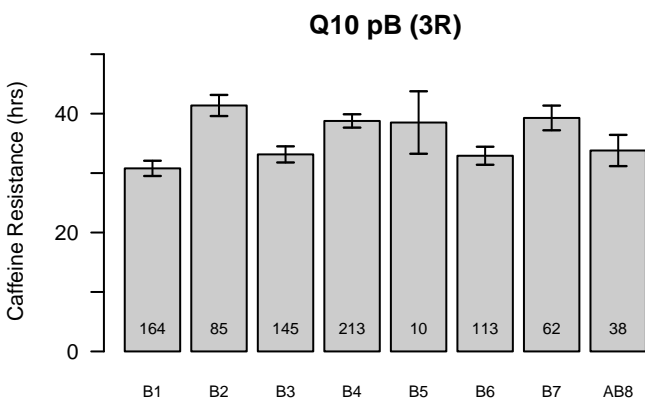

Supplement: S3 Fig — The number of RILs for which we assign a founder genotype (probability > 0.95) is listed at the bottom of each bar. Only founder means associated with at least 5 observations are presented. (PDF) [file pgen.1005663.s003.pdf]

## Effect of Cyp12d1 CNV on Caffeine Resistance

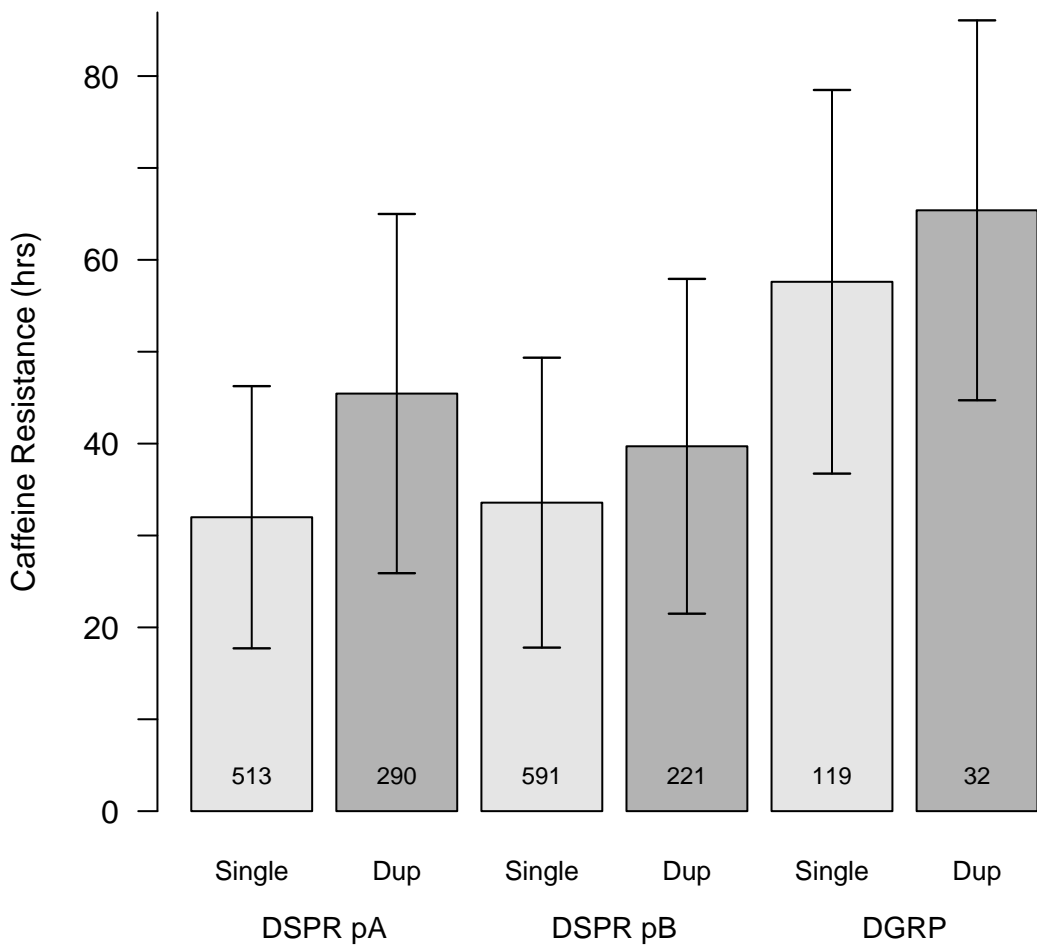

Supplement: S4 Fig — The plots show the mean phenotype (± 1-SD) of those lines with one copy of Cyp12d1 ("single") or two copies ("Dup"), with the number of lines in each class shown at the bottom of each bar. There is a significant effect of the duplication on caffeine resistance in the DSPR pA population (Welch's t-test, p < 10−15) and in the DSPR pB population (Welch's t-test, p < 10−5). However, the effect on the DGRP is not formally significant (Welch's t-test, p = 0.065). (PDF) [file pgen.1005663.s004.pdf]

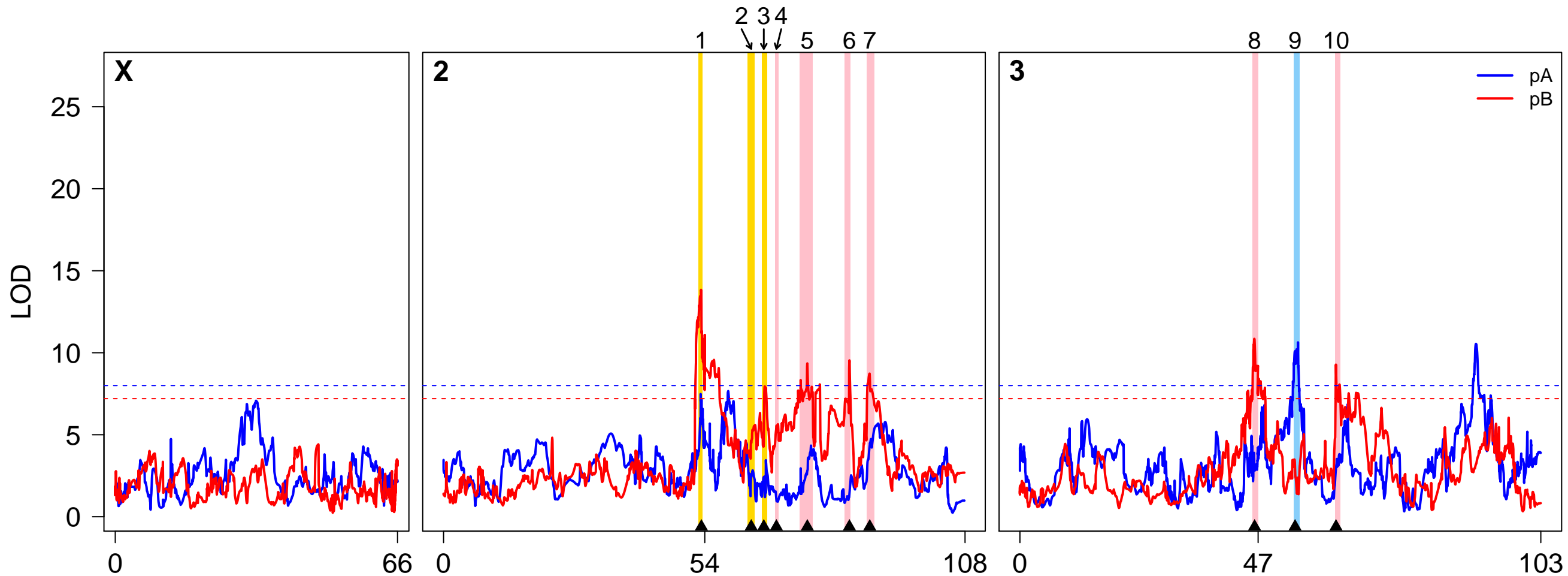

Supplement: S5 Fig — The format of this plot is identical to that of Fig 1 in the main text, but with genomewide 5% permutation thresholds of 8.0 LOD (pA) and 7.2 LOD (pB). In the original genome scan we identified 10 QTL, three in both populations (Q1, Q2, Q3), one in pA only (Q9), and six in pB only (Q4, Q5, Q6, Q7, Q8, Q10). After controlling for the CNV, we continue to identify Q1 in both panels, Q9 in pA only, and Q5, Q6, Q7, Q8, and Q10 in pB only. Q2—the QTL that harbors Cyp12d1—disappears in both panels, suggesting much of the effect of that QTL is due to the CNV and/or factors in LD with this variant. Q3 is now only identified in pB, and is absent in pA. The peak at Q4 is also absent. Finally, a peak towards the end of 3R in pA (3R:21960000..22250000), that was just below the significance threshold in our original analysis, is now significant. (PDF) [file pgen.1005663.s005.pdf]

# Effect of CNV on Cyp12d1 Expression in Female Heads

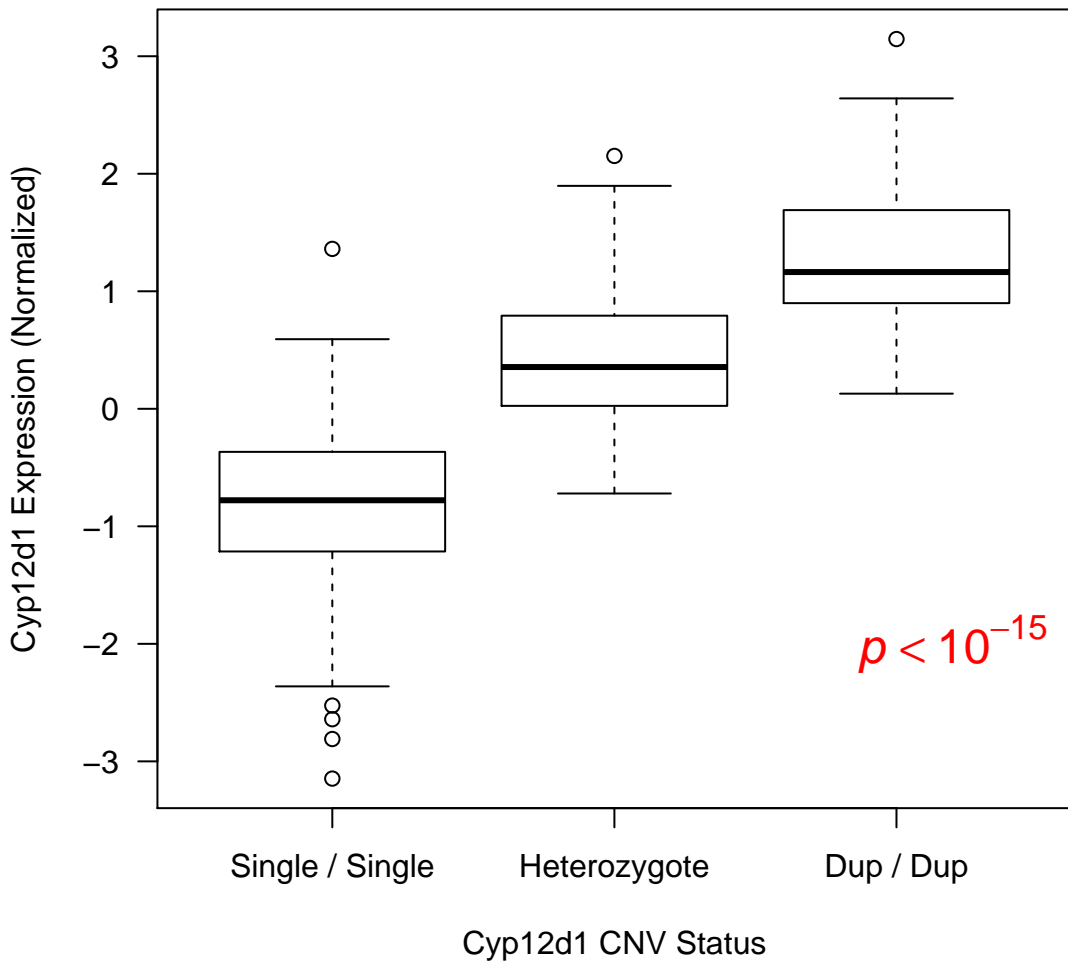

Supplement: S6 Fig — King et al. [36] generated array-based, genomewide expression data from female head tissue for 600 genotypes. Each genotype was the result of a cross between an independent pair of DSPR RILs. Using the normalized expression data for Cyp12d1, which represents a composite measure of expression from any and all gene copies present in the target genotype since the gene copies are not distinguished on the array, and the CNV status provided in the current study, there is a strong effect of CNV status on Cyp12d1 expression (linear regression, p < 10−15). (PDF) [file pgen.1005663.s006.pdf]
