# Supplementary material for: Identifying Loci Contributing to Natural Variation in Xenobiotic Resistance in Drosophila
Source: PLoS Genet. 2015 Nov 30;11(11):e1005663. doi: 10.1371/journal.pgen.1005663 (PMC4664282; doi:10.1371/journal.pgen.1005663)
Supplement: S1 Dataset — Data is taken directly from the "gene_exp.diff" Cuffdiff output file. (PDF) [file pgen.1005663.s007.pdf]

Dataset S1. Expression levels of the five P450 genes under mapped QTL. Data is taken directly from the "gene_exp.diff" Cuffdiff output file.

QTL:	Q1
Gene:	*Cyp310a1*
Low-control FPKM:	0.6690
High-control FPKM:	0.5216
Low-caffeine FPKM:	0.5971
High-caffeine FPKM:	0.5448
Tests:	No tests significant at $p < 0.05$

QTL:	Q2
Gene:	*Cyp12d1*
Low-control FPKM:	26.3
High-control FPKM:	102.3
Low-caffeine FPKM:	293.3
High-caffeine FPKM:	700.8
Tests:	See Table 2

QTL:	Q3
Gene:	*Cyp301a1*
Low-control FPKM:	1.837
High-control FPKM:	1.880
Low-caffeine FPKM:	1.955
High-caffeine FPKM:	2.242
Tests:	No tests significant at $p < 0.05$

QTL:	Q9
Gene:	*Cyp313a1*
Low-control FPKM:	18.62
High-control FPKM:	12.14
Low-caffeine FPKM:	7.91
High-caffeine FPKM:	8.19
Tests:	Low-control versus Low-caffeine ($p = 0.005$)<br>All other tests do not reach significance at $p < 0.05$

QTL:	Q2
Gene:	*Cyp6d5*
Low-control FPKM:	85.3
High-control FPKM:	175.8
Low-caffeine FPKM:	737.3
High-caffeine FPKM:	768.4
Tests:	See Table 2
