# Supplementary material for: Identifying Loci Contributing to Natural Variation in Xenobiotic Resistance in Drosophila
Source: PLoS Genet. 2015 Nov 30;11(11):e1005663. doi: 10.1371/journal.pgen.1005663 (PMC4664282; doi:10.1371/journal.pgen.1005663)
Supplement: S2 Dataset — All data is taken directly from VDRC website (stockcenter.vdrc.at; Accessed January 8, 2015). Hairpin sequences designed to target one member of the gene pair have strong similarity with sequence from the other gene. Bases in hairpin sequences that are different between the two genes are highlighted in green. (PDF) [file pgen.1005663.s008.pdf]

Dataset S2. Vienna *Drosophila* Resource Center (VDRC) UAS-RNAi hairpin sequences for *Cyp12d1-d* and *Cyp12d1-p* genes. All data below taken directly from VDRC website (stockcenter.vdrc.at; Accessed January 8, 2015). Hairpin sequences designed to target one member of the gene pair have strong similarity with sequence from the other gene. Bases in hairpin sequences that are different between the two genes are highlighted in green.

Target Gene: *Cyp12d1-d*
VDRC Transformant ID: 50507
Library Type: GD
Hairpin Length: 306

Hairpin Sequence:

```
cgcgatctgt ggcgatttac gtgggtcccg ttcgatcttc aagatcagcc tccgttttgg cacatgaaca
agctaaatct agtataacgg aggagcacaa gacctacgat gagattccgc ggcccaacaa attcaaattt
atgagggctt tcatgcccgg tggtgaattc caaaatgcat cgattacgga atacaccagt gccatgcgaa
agcgctatgg agatatctat gtaatgcccg gaatgtttgg ccgcaaggat tgggtcacca ctttcaacac
aaaggacatt gagatggtat tccgca
```

Sequence perfectly matches both *Cyp12d1-d* and *Cyp12d1-p*.

Target Gene: *Cyp12d1-p*
VDRC Transformant ID: 21235
Library Type: GD
Hairpin Length: 367

Hairpin Sequence:

```
ccacgcttct gtcggctgtt ttactctgcc tctcaaagca tccggataag caggccaaac tgcgggagga
gctcttgagc atcatgccca caaaggattc cctactcaac gaggagaaca tgaaggatat gccatatttg
agggctgtga tcaaggagac actgcgatac tatcccaatg gcttGggaac catgagaacc tgccaaaatg
atgtgatact ttcgggttac cgggtgccca agggaacgac tgtcctgctc ggctcaaatg tgctgatgaa
ggaagctaca tattatccac gaccagatga atttctgccg gagcgctggt taagggatcc ggagaccgga
aagaagatgc aggtcag
```

Sequence perfectly matches *Cyp12d1-p*, and is 1 nucleotide different from *Cyp12d1-d*.

Target Gene: *Cyp12d1-d*
VDRC Transformant ID: 109248
Library Type: KK
Hairpin Length: 177

Hairpin Sequence:

```
ctcataatca ccgccacctt gggatcgatc tccattaatc tctccagcat actgttacta tttatttttt
cgccagcctg gcggcgcttt tccagtgcat cctgattttc cttcagcatt ttctgggAca cattcaaact
gtcattgaga gttcgcttca ttttcctgta ggtgggc
```

Sequence perfectly matches *Cyp12d1-d*, and is 1 nucleotide different from *Cyp12d1-p*.

Target Gene: *Cyp12d1-p*
VDRC Transformant ID: 109256
Library Type: KK
Hairpin Length: 82

Hairpin Sequence:
Cgaacatggt cttgaatgga cggctggcat cccgattgaa ctccacatgg aaattgcgaa tcaacttggc
cactgtggtc tc

Sequence perfectly matches *Cyp12d1-p*, and is 1 nucleotide different from *Cyp12d1-d*.

Target Gene: *Cyp12d1-p*
VDRC Transformant ID: 49269
Library Type: GD
Hairpin Length: 388

Hairpin Sequence:
ccaatggctt Gggaaccatg agaacctgcc aaaatgatgt gatactttcg ggttaccggg tgcccaaggg
aacgactgtc ctgctcggct caaatgtgct gatgaaggaa gctacatatt atccacgacc agatgaattt
ctgccggagc gctggttaag ggatccggag accggaaaga agatgcaggt cagccccttc acgttccttc
cctttggctt tggaccccgc atgtgcattg gcaaacgggt ggtggatctg gaaatggaga ccacagtggc
caagttgatt cgcaatttcc atgtggagtt caatcgggat gccagccgtc cattcaagac catgttcGtc
atggaaccgg ccattacgtt ccccttcaaa ttcacgga

Sequence perfectly matches *Cyp12d1-p*, and is 2 nucleotides different from *Cyp12d1-d*.
