# Supplementary material for: Identifying Loci Contributing to Natural Variation in Xenobiotic Resistance in Drosophila
Source: PLoS Genet. 2015 Nov 30;11(11):e1005663. doi: 10.1371/journal.pgen.1005663 (PMC4664282; doi:10.1371/journal.pgen.1005663)
Supplement: S3 Dataset — (PDF) [file pgen.1005663.s009.pdf]

Dataset S3. Copy number variation (CNV) genotype calls for the *Cyp12d1* gene region in the DSPR and DGRP mapping panels.

<u>DSPR founders</u>

Founders with one copy of *Cyp12d1*:

A1, A2, A3, A4, A5, A6, AB8, B2, B3, B4, B6

Founders with two copies of *Cyp12d1*:

A7, B1, B5, B7

<u>DGRP inbred lines</u>

Strains with one copy of *Cyp12d1*:

25174, 25175, 25176, 25181, 25182, 25183, 25184, 25185, 25186, 25187, 25189, 25191, 25192, 25194, 25195, 25198, 25200, 25201, 25202, 25203, 25205, 25206, 25209, 28123, 28124, 28125, 28126, 28127, 28128, 28130, 28131, 28132, 28134, 28135, 28137, 28139, 28141, 28143, 28144, 28145, 28146, 28147, 28148, 28149, 28151, 28152, 28154, 28155, 28156, 28157, 28159, 28160, 28162, 28164, 28166, 28170, 28171, 28173, 28174, 28176, 28177, 28178, 28179, 28180, 28182, 28183, 28185, 28186, 28191, 28192, 28194, 28196, 28199, 28204, 28208, 28211, 28212, 28213, 28215, 28220, 28221, 28222, 28223, 28224, 28226, 28230, 28231, 28235, 28236, 28238, 28239, 28241, 28243, 28244, 28246, 28247, 28249, 28250, 28251, 28252, 28253, 28254, 28255, 28257, 28259, 28260, 28261, 28262, 28274, 28276, 28278, 29652, 29653, 29654, 29655, 29657, 29659, 29660, 37525

Strains with two copies of *Cyp12d1*:

25179, 25190, 25207, 25445, 25745, 28129, 28136, 28140, 28150, 28161, 28167, 28168, 28184, 28188, 28189, 28190, 28200, 28202, 28207, 28216, 28219, 28227, 28232, 28233, 28234, 28240, 28245, 28256, 28258, 28263, 28265, 29656
