# Supplementary material for: Identifying Loci Contributing to Natural Variation in Xenobiotic Resistance in Drosophila
Source: PLoS Genet. 2015 Nov 30;11(11):e1005663. doi: 10.1371/journal.pgen.1005663 (PMC4664282; doi:10.1371/journal.pgen.1005663)
Supplement: S1 Text — (PDF) [file pgen.1005663.s010.pdf]

Text S1. Code used for RNAseq analysis.

```
# Sickle (version 1.200)
# Run on each of the four FASTQ files:
#    Low-control (Lcon), Low-caffeine (Lcaff), High-control (Hcon), High-caffeine (Hcaff)

sickle se -f Lcon.fastq.gz -t sanger -o Lcon.sickle.fastq -q 30 -l 30 -n
gzip Lcon.sickle.fastq


# TopHat (version 2.0.9)
# Run on each of the four trimmed FASTQ files

tophat2 -p 8 -G genes.gtf -o ./Assembly_Lcon/ --no-novel-juncs --library-type
   fr-unstranded genome Lcon.sickle.fastq.gz


# Cufflinks (version 2.1.1)
# Run on all four TopHat assemblies simultaneously

cuffdiff -N -o ./CuffDiff_Output/ -b genome.fa -p 12 -u genes.gtf
   -L Lcon,Lcaff,Hcon,Hcaff ./Assembly_Lcon/accepted_hits.bam
   ./Assembly_Lcaff/accepted_hits.bam ./Assembly_Hcon/accepted_hits.bam
   ./Assembly_Hcaff/accepted_hits.bam
```
